# Molecular Characterization of Soritesidine Reveals a Novel Marine Sponge DNase I-like Toxin

**DOI:** 10.3390/toxins18100434

**Published:** 2026-10-09

**Authors:** Hiromi Watari, Koji Nakano, Masaya Kitano, Hiromu Kageyama, Yoshikazu Tanaka, Yuri Ishibashi, Ryuichi Sakai

**Affiliations:** 1 Faculty and Graduate School of Fisheries Sciences, Hokkaido University, Hakodate 041-8611, Japan; 2 Graduate School of Life Sciences, Tohoku University, Sendai 980-8578, Japanyoshikazu.tanaka@tohoku.ac.jp (Y.T.)

**Keywords:** sponge, DNase I. cytolethal distending toxin, protein

## Abstract

Soritesidine (SOR) is a potent proteinaceous toxin from the marine sponge *Spongosorites* sp. that is toxic to mammalian cells, brine shrimp larvae, sea hare embryos, and mice. Although SOR exhibits divalent metal ion-dependent DNase activity, its molecular identity and mechanism of action remained unknown. We determined the amino acid sequence of SOR and investigated its cellular mode of action. Degenerate PCR, 3′-RACE, inverse PCR, and nested PCR identified an open reading frame encoding a 947-amino-acid acidic protein. The deduced sequence showed no substantial similarity to known proteins except for approximately 30% sequence identity to a hypothetical protein from the marine α-proteobacterium *Euryhalocaulis caribicus*. Analysis of the AlphaFold 3-predicted N-terminal domain using Foldseek and structural superposition revealed a DNase I-like fold with a catalytic architecture similar to that of vertebrate DNase I. A recombinant N-terminal fragment (residues 1–310, N-SOR) exhibited DNA cleavage activity, supporting the assignment of this region as a DNase I-like endonuclease domain. The recombinant fragment lacked detectable cytotoxicity, suggesting that regions outside the N-terminal DNase I-like domain contribute to the cytotoxicity of SOR. Fluorescence imaging showed that SOR entered cells and accumulated in the nucleus, whereas its cellular uptake was attenuated at 4 °C, suggesting the involvement of an energy-dependent internalization pathway. Immunostaining revealed increased γH2AX signals in the nuclei of SOR-treated cells, indicating activation of a DNA damage response. Together, these findings identify SOR as an unusual multidomain protein toxin containing a functional DNase I-like domain and provide a molecular basis for further investigation of its cytotoxic mechanism.

## 1. Introduction

Marine sponges (phylum Porifera) are among the richest sources of natural products, with more than 20,000 secondary metabolites reported to date. Many of these compounds exhibit potent cytotoxic, antimicrobial, antiviral, and anti-inflammatory activities and have served as valuable leads for drug discovery. In contrast to these extensively studied small molecules, sponge proteins remain largely unexplored despite their remarkable structural diversity and functional potential [1,2]. Increasing evidence, however, suggests that sponge proteins constitute a unique class of bioactive molecules whose mechanisms of action are fundamentally different from those of conventional secondary metabolites [1]. Our recent studies have revealed remarkably diverse biological functions among sponge-derived proteins. These include the glutamate receptor-modulating lectin CchG [3], the thrombopoietin receptor agonist (ThC) [4], and the cell-penetrating lectin RaspL [5], demonstrating that marine sponge proteins can regulate receptor signaling, cellular uptake, and intracellular trafficking through mechanisms not previously observed in marine natural products.

Protein toxins are among the most sophisticated biological molecules evolved in nature. Classical bacterial toxins employ diverse catalytic mechanisms to disrupt essential cellular processes. Examples include botulinum neurotoxin [6], diphtheria toxin [7], and cholera toxin [8], as well as genotoxins such as cytolethal distending toxin (CDT) [9], whose catalytic subunit functions as DNase I to induce DNA damage following intracellular delivery. These toxins have greatly advanced our understanding of cell biology and have also become valuable therapeutic agents and research tools.

Compared with bacterial protein toxins, however, cytotoxic proteins from marine sponges remain poorly understood. Only a limited number of sponge-derived protein toxins have been reported, and their molecular structures and mechanisms of action remain largely unknown [10]. The discovery of novel sponge protein toxins therefore offers an opportunity to uncover previously unrecognized strategies for cellular intoxication and may provide new molecular tools for biological and biomedical research.

In our previous study, we isolated soritesidine (SOR) from the marine sponge *Spongosorites* sp. SOR is an acidic protein with a molecular mass of approximately 109 kDa and exhibits remarkable toxicity toward cultured mammalian cells, brine shrimp larvae, sea hare embryos, and mice. Partial amino acid sequencing failed to identify any homologous proteins, suggesting that SOR represents a previously undescribed protein family. Interestingly, SOR also exhibited divalent metal ion-dependent DNase activity, raising the possibility that it acts through a mechanism analogous to bacterial DNase-containing genotoxins [11]. However, neither its complete amino acid sequence nor its mechanism of cellular intoxication had been elucidated.

In the present study, we determined the complete amino acid sequence of SOR by molecular cloning and identified a previously unrecognized DNase I-like catalytic domain. We further demonstrate that SOR enters cells through an endocytosis-dependent pathway and accumulates in the nucleus, where its DNase I-like domain is likely to exert its cytotoxic activity. These findings establish SOR as a previously undescribed marine sponge protein toxin and further illustrate the remarkable functional diversity of sponge-derived proteins.

## 2. Results

### 2.1. Amino Acid Sequence of SOR

**Figure 1:**
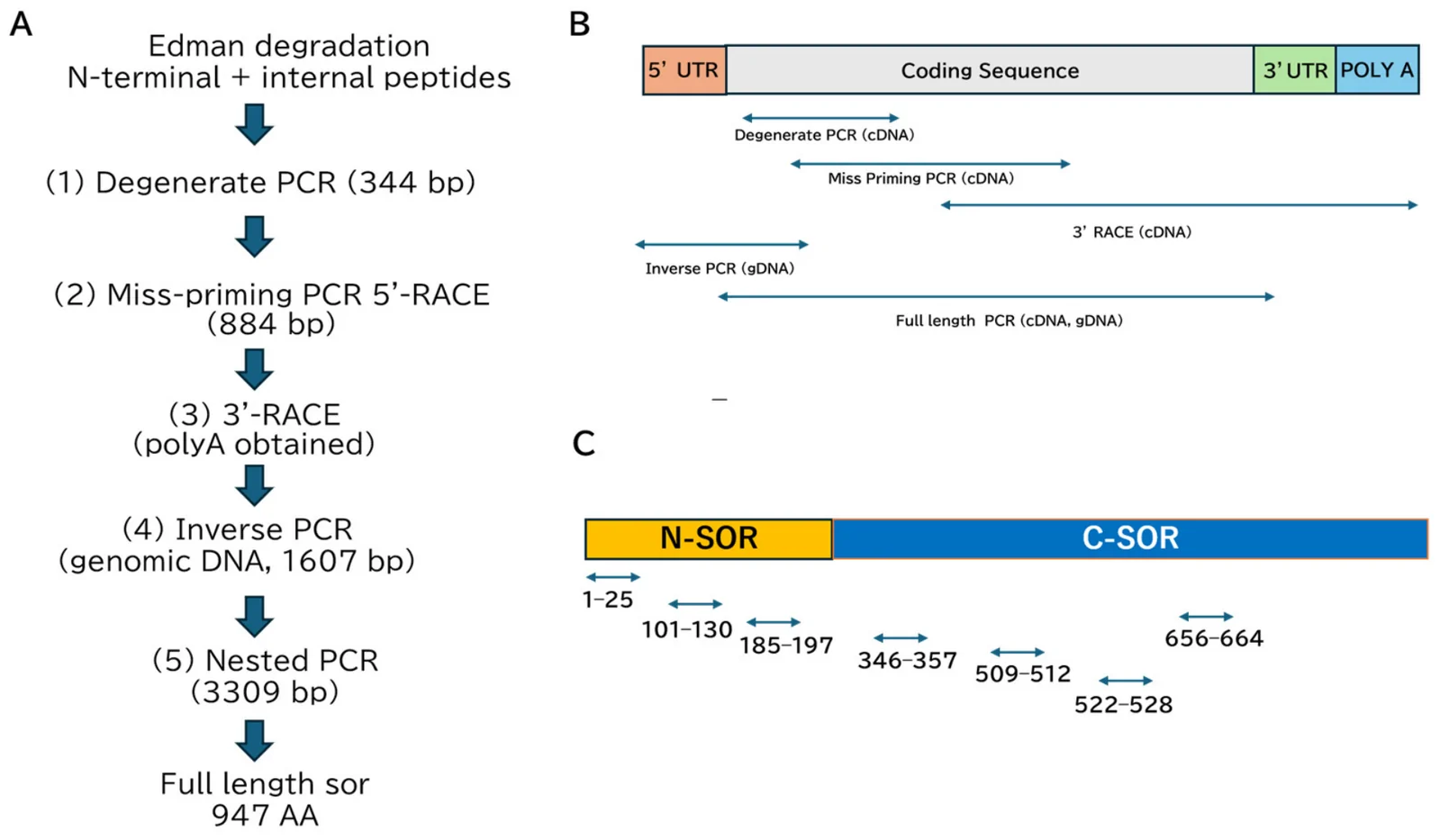
An overview of cDNA and genomic DNA analysis of *sor*. (**A**) Workflow for cloning the full-length *sor* gene. (**B**) Assembly of the sor coding sequence from overlapping PCR fragments and domain organization of SOR. The start codon was not detected by the inverse PCR experiment of genomic DNA. (**C**) Domain organization of SOR. Arrows indicate positions of peptide fragments identified by Edman degradation.

We have determined the N-terminal 25-amino acid sequence and internal amino acid sequences of SOR by Edman degradation [11]. A total of 20 peptide fragments composed of 160 amino acids were determined (Table S1). We next turned to deducing the whole amino acid sequence of SOR from the nucleic acid sequence of the gene denoted *sor* (Figure 1). A cDNA library was prepared from mRNA extracted from the sponge specimen. Degenerate primers were designed based on the N-terminal amino acid sequence and some internal sequence obtained above (Table S2). The first-strand PCR amplified a 345 bp gene that encodes an N-terminal 115-amino-acid peptide (Figure S1). We next attempted to obtain the entire sequence by 3’-RACE using the above information; however, the expected polyA at the 3’ end was absent. Instead, an 884 bp fragment was amplified (Figure S2A), which encoded several internal peptide sequences previously identified by Edman degradation and therefore appeared to represent a portion of the *sor* gene (Figure S2B).

Detailed sequence analysis revealed that this fragment had been generated through an unusual PCR event. The sequence at the 3′ end of the target region happened to be complementary to the forward primer, allowing the same primer to anneal at both ends of the amplified region. Consequently, the 884 bp product was generated through unintended self-priming by the forward primer. We refer to this phenomenon as “mis-priming PCR” (Figure S2C).

Although obtained fortuitously, the mis-priming product proved highly useful for subsequent gene cloning. Sequence information derived from this fragment enabled the design of new gene-specific primers, and subsequent 3′-RACE experiments successfully yielded four independent clones containing a poly(A) tail at the 3′ end of the sor transcript (Figure S3A,B).

We next attempted to determine the upstream 5′-end sequence of sor. Initial 5′-RACE experiments using first-strand cDNA as a template failed to yield any PCR product. We therefore adopted an inverse PCR strategy using genomic DNA as a template (Figure S4A). This approach was based on our previous finding that the sponge lectin gene *cchG* is encoded directly in genomic DNA without introns [3].

Genomic DNA was digested with PstI, self-ligated to generate circular DNA molecules, and used as a template for inverse PCR [12]. This procedure yielded a 1607 bp PCR product (Figure S4B). Sequence analysis showed that the amplified fragment overlapped well with the sequences obtained from both the first-strand PCR and the mis-priming PCR experiments. Surprisingly, no PstI recognition site was found within the amplified region. Although the reason for this remains unclear, this observation suggested that sor may also be encoded directly in the genomic DNA.

To confirm the complete sequence of sor, nested PCR was performed using primers designed based on the 3′-RACE and inverse PCR products. Using first-strand cDNA as a template, fourteen independent clones containing a 3309 bp PCR product were obtained. In addition, four clones of the same size were amplified directly from genomic DNA. Sequence comparison between these clones and the 3’-RACE product showed that all clones were highly similar except for two nucleotide substitutions identified in the full-length PCR products, which resulted in amino acid replacements Y428N and L653P.

By assembling the overlapping sequences obtained from first-strand PCR, mis-priming PCR, 3′-RACE, inverse PCR, and nested PCR experiments, a complete open reading frame encoding a 947-amino-acid protein was deduced for SOR (Figure S5).

The theoretical molecular weight was calculated to be 103,911, smaller by approximately 5 kDa than that of the native protein determined by MALDI-TOFMS. The isoelectric point was calculated to be 4.83, which agreed well with that observed (4.5) [11].

Interestingly, the full-length amino acid sequence of SOR showed approximately 30% sequence identity to a hypothetical protein from the marine α-proteobacterium *E. caribicus* (GenBank accession no. WP_022696775). While this finding may indicate an evolutionary relationship between SOR and bacterial proteins, the presence of a polyadenylated sor transcript strongly supports the eukaryotic origin of the *sor* gene and is consistent with SOR being a sponge-derived protein. However, other eukaryotic sources, such as symbiotic fungi or microalgae, cannot be excluded at present.

**Figure 2:**
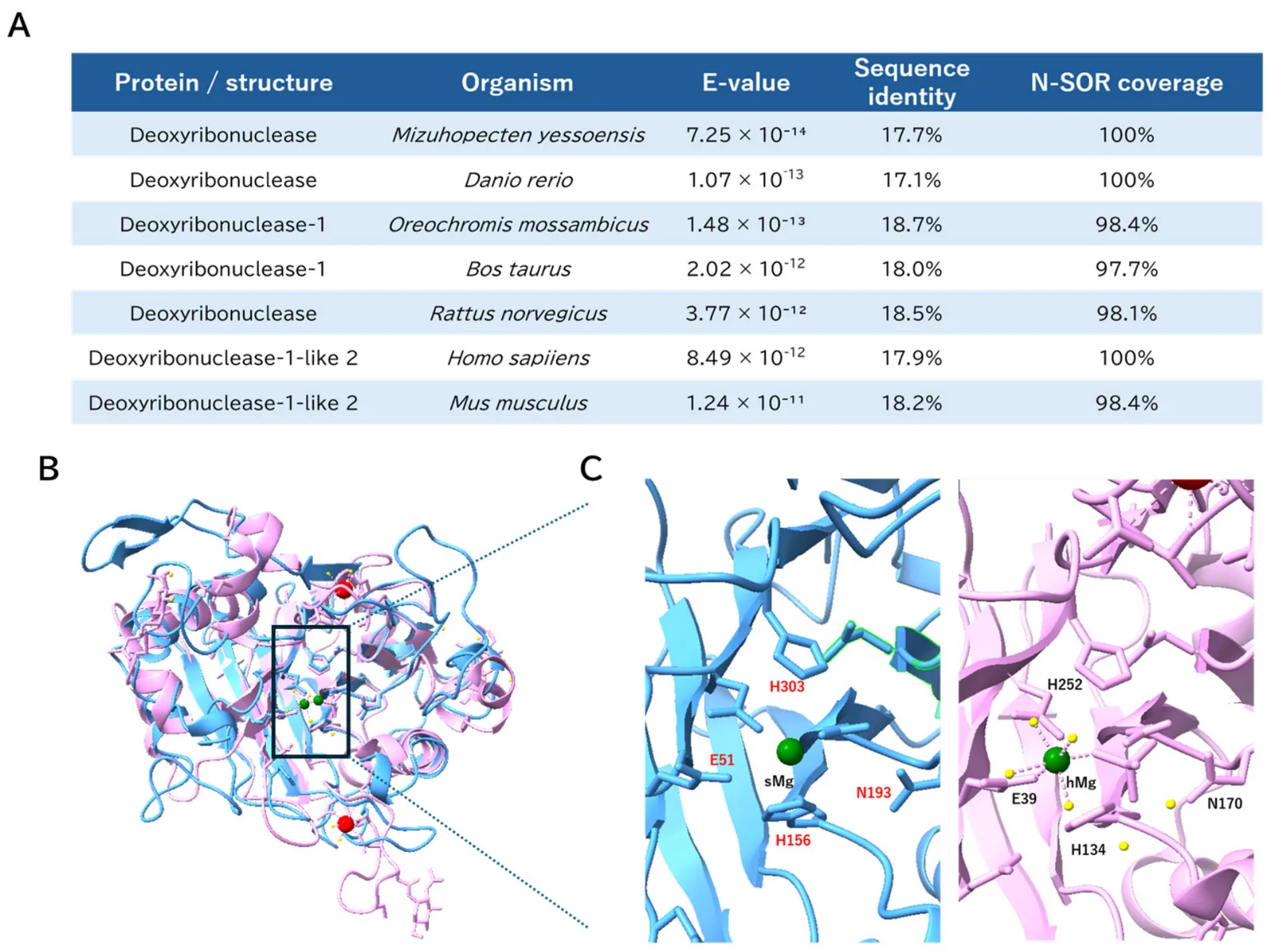
Structural features of the N-terminal domain of SOR (N-SOR). (**A**) Structural similarity search of N-SOR (residues 1–310) using the AlphaFold3-predicted structure as the query in Foldseek. The seven highest-scoring structural homologs are listed. (**B**) A comparison of the predicted structure of N-SOR (blue) and the crystal structure of human DNase I (PDB ID: 4AWN, pink) [14], with (**C**) a close-up view of the catalytic center. The Mg^2+^ ions in N-SOR (sMg) and human DNase I (hMg) are shown in green, and water molecules are shown as yellow spheres. Representative catalytic residues are labeled in red (N-SOR) and black (human DNase I).

Foldseek analysis of the AlphaFold 3-predicted structure of the N-terminal domain revealed significant structural similarity to vertebrate DNase I proteins (Figure 2A). Structural comparison further showed that residues corresponding to the metal-binding catalytic site of human DNase I occupy similar spatial positions in the SOR model, consistent with the experimentally observed DNase activity (Figure 2). The N-terminal region of SOR (residues 8–310) could be aligned with residues 1–259 of bovine DNase I, with approximately 20% sequence identity.

To further examine the structural organization of SOR, the full-length AlphaFold 3 model was divided into four predicted structural domains and individually analyzed using Foldseek (Figure S6). In addition to the N-terminal DNase I-like domain, domain 3 (residues 462–808) showed strong structural similarity to proteins annotated as endonuclease/exonuclease/phosphatase family proteins, with the highest-ranking matches covering nearly the entire domain. Domain 2 also showed significant structural similarities to several structurally diverse proteins, including Ig-like domain-containing proteins, whereas no strongly significant structural homolog was identified for domain 4. These results suggest that SOR has a complex multidomain architecture, although the biological functions of the regions outside the experimentally characterized N-terminal DNase I-like domain remain unclear. SOR represents an unusual protein toxin in which a DNase I-like domain is incorporated into a single polypeptide. In comparison, cytolethal distending toxins (CDTs), a well-characterized family of bacterial genotoxins, contain a DNase I-like catalytic subunit together with two additional subunits involved in cell binding, internalization, and intracellular trafficking [13].

### 2.2. Production and Biological Evaluation of Recombinant SOR

We produced recombinant SOR (rSOR) in *E. coli* in an insoluble, non-toxic form (Figure S7A). We also produced a recombinant theoretical protein found in the genome of the marine α-proteobacterium *Euryhalocaulis caribicus* (ECP), which has 30% homology to SOR (Figure S7B). As expected, both proteins produced as insoluble form were not bioactive and several refolding experiments failed to solubilize them.

**Figure 3:**
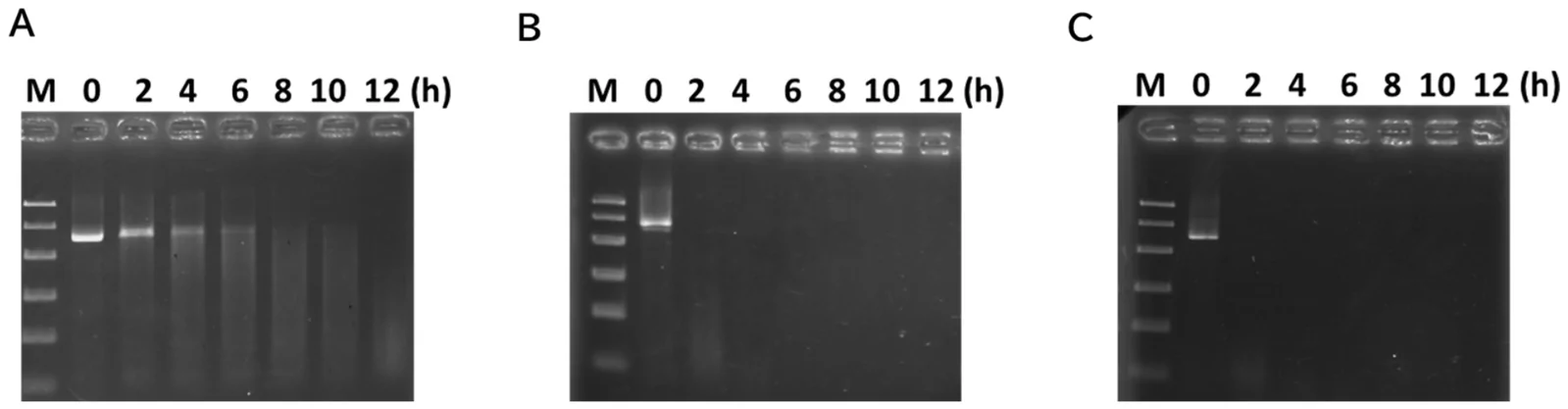
DNA cleavage assay of (**A**) recombinant N-SOR, (**B**) native SOR and (**C**) bovine DNase I. The concentration was adjusted to approximately 0.8–1μM. DNA was prepared by PCR amplification of the PBS SK(+) plasmid (~3000 bp) and incubated with each enzyme for up to 12 h at pH 8.

We therefore turned our attention to the N-terminal DNase I moiety of SOR (N-SOR), which is likely responsible for the DNA-cleaving activity of SOR suggested in our previous study [11]. To further confirm this idea, we prepared recombinant N-SOR. The synthetic gene (*rN-sor*, 930 bp, optimized for *E. coli*; Figure S8A) encoding an N-terminal 310-amino-acid residue was expressed in *E. coli* in soluble form. The N-terminal 310-amino-acid region of SOR was expressed in *E. coli* predominantly as an insoluble protein (Figure S8B). Ni^2+^-affinity purification under denaturing conditions followed by refolding yielded a protein with DNase activity (Figure 3). Although N-SOR cleaved DNA at a molar concentration comparable to those of bovine DNase I and native SOR, a considerably longer reaction time was required to achieve comparable DNA cleavage. The cytotoxicity of N-SOR was compared with that of native SOR using Ba/F3 murine cells. N-SOR did not affect cell proliferation at the highest concentration tested (3.3 μg/mL), while SOR inhibited 60% of cell proliferation at 80 pg/mL.

### 2.3. Cellular Translocation of SOR

The above results suggest that SOR contains an N-terminal endonuclease domain that functions as the catalytic warhead, while the N-terminal region alone is not sufficient for exhibiting cellular toxicity.

We next investigated the cellular translocation and intracellular trafficking of SOR using FITC-labeled SOR (FITC-SOR). Because recombinant SOR could not be refolded into its native conformation, native SOR purified by size-exclusion chromatography was used for FITC labeling. FITC-labeled SOR retained potent biological activity (IC_50_ approximately 0.25 ng/mL), although its cytotoxicity was reduced compared with that of the native protein (0.06 ng/mL) [11], indicating that FITC labeling attenuated but did not abolish its activity (Figure S9). FITC-SOR was therefore used for the following cellular localization studies.

**Figure 4:**
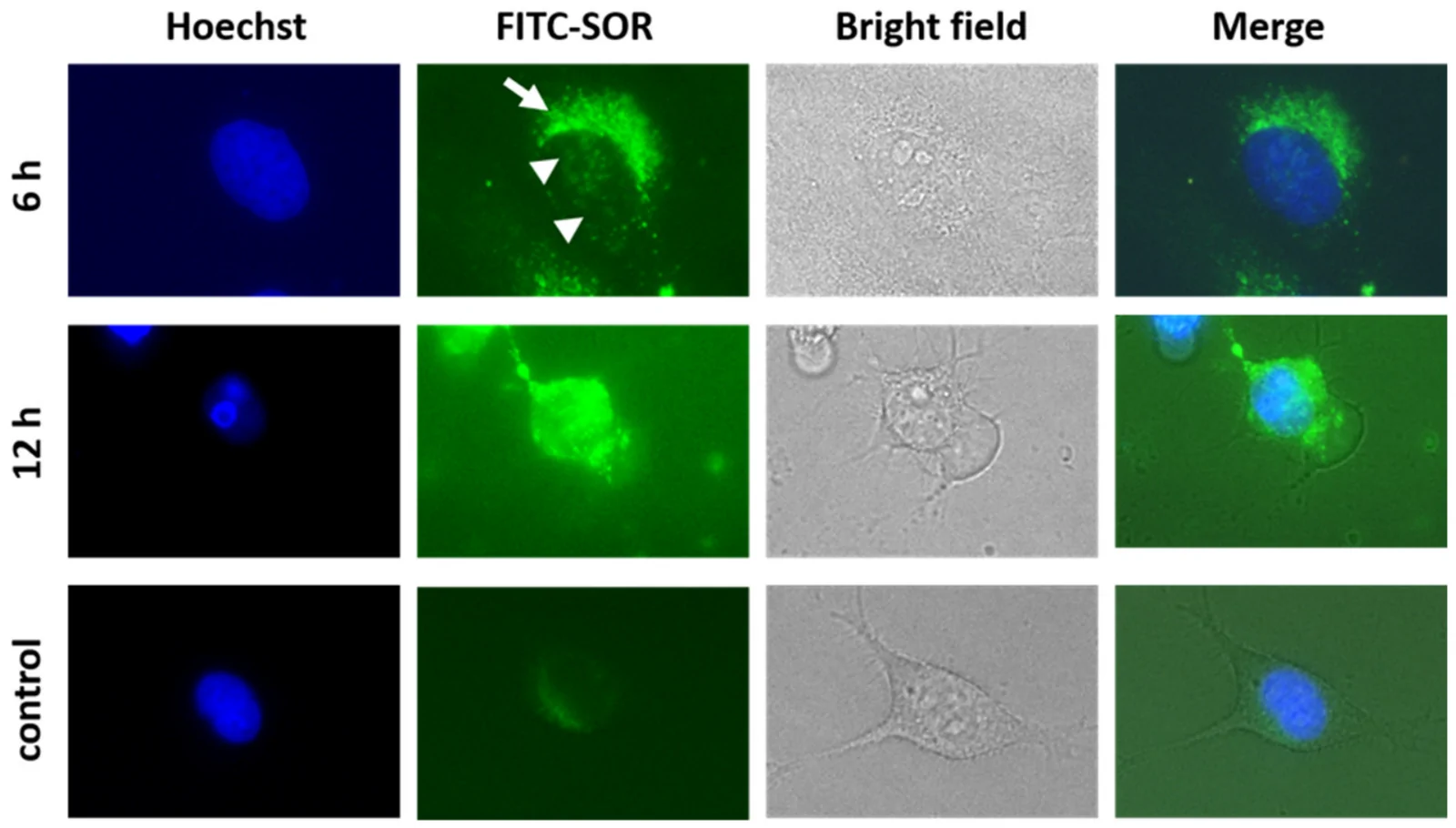
Fluorescence micrographs of HeLa cells treated with FITC-SOR (10 μg/mL). Fluorescence images showing FITC-SOR (green), Hoechst 33258 (blue), corresponding transmitted bright-field images, and merged fluorescence images. At 6 h after treatment, FITC-SOR was predominantly localized in the perinuclear region (white arrow), although weak nuclear fluorescence was also observed in some cells (arrowheads). By 12 h, most cells exhibited marked shrinkage together with severe disruption of the cytoplasm and intracellular organelles.

HeLa cells treated with FITC-labeled SOR (FITC-SOR, 10 μg/mL) were examined by fluorescence microscopy at 6 and 12 h after treatment. At 6 h, FITC fluorescence was detected predominantly in the perinuclear region, consistent with localization in the endoplasmic reticulum (ER) and Golgi apparatus, while nuclear morphology remained largely intact. Notably, weak but distinct FITC fluorescence was also observed within the nucleus (Figure 4). By 12 h, marked morphological changes had developed, including cell shrinkage, chromatin condensation, and nuclear fragmentation, and many cells had detached from the culture surface.

When FITC-SOR treatment was performed at 4 °C, fluorescence was predominantly detected at the cell surface, with little or no intracellular or nuclear fluorescence. In contrast, clear intracellular and nuclear fluorescence was observed at 37 °C (Figure S10). Together, these results demonstrate that SOR is internalized into HeLa cells in an energy-dependent manner, accumulates initially in the perinuclear region, and subsequently translocates into the nucleus.

### 2.4. Cellular Response After SOR Treatment

**Figure 5:**
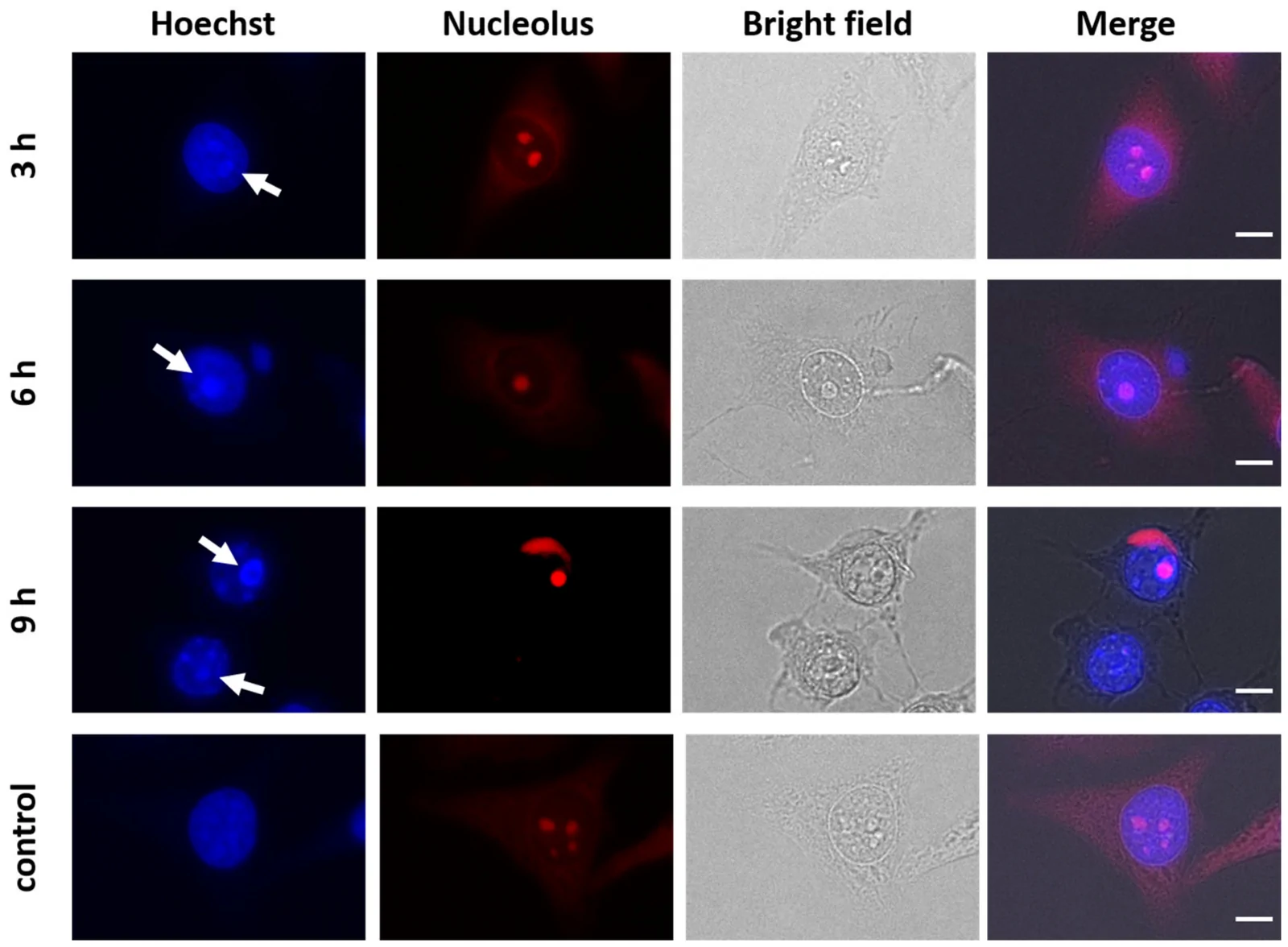
Time-dependent morphological changes in HeLa cells treated with SOR. Fluorescence images showing Hoechst 33258-stained nuclei (blue), a Nucleolus Bright Red-stained nucleolus (red), corresponding transmitted bright-field images, and merged fluorescence images. HeLa cells were treated with SOR (100 ng/mL) and observed at the indicated time points. Chromatin condensation became apparent 3 h after SOR treatment and progressed around the nucleolus by 6 h. By 9 h, most cells had lost their structural integrity, while fluorescence became markedly concentrated in the nucleolus. Scale bar = 10 μm.

To investigate the cellular responses induced by SOR, HeLa cells were observed for 9 h under a fluorescence microscope with nuclear and nucleolus staining (Figure 5). To ensure short-term observation, a high concentration (100 ng/mL) of SOR was used.

**Figure 6:**
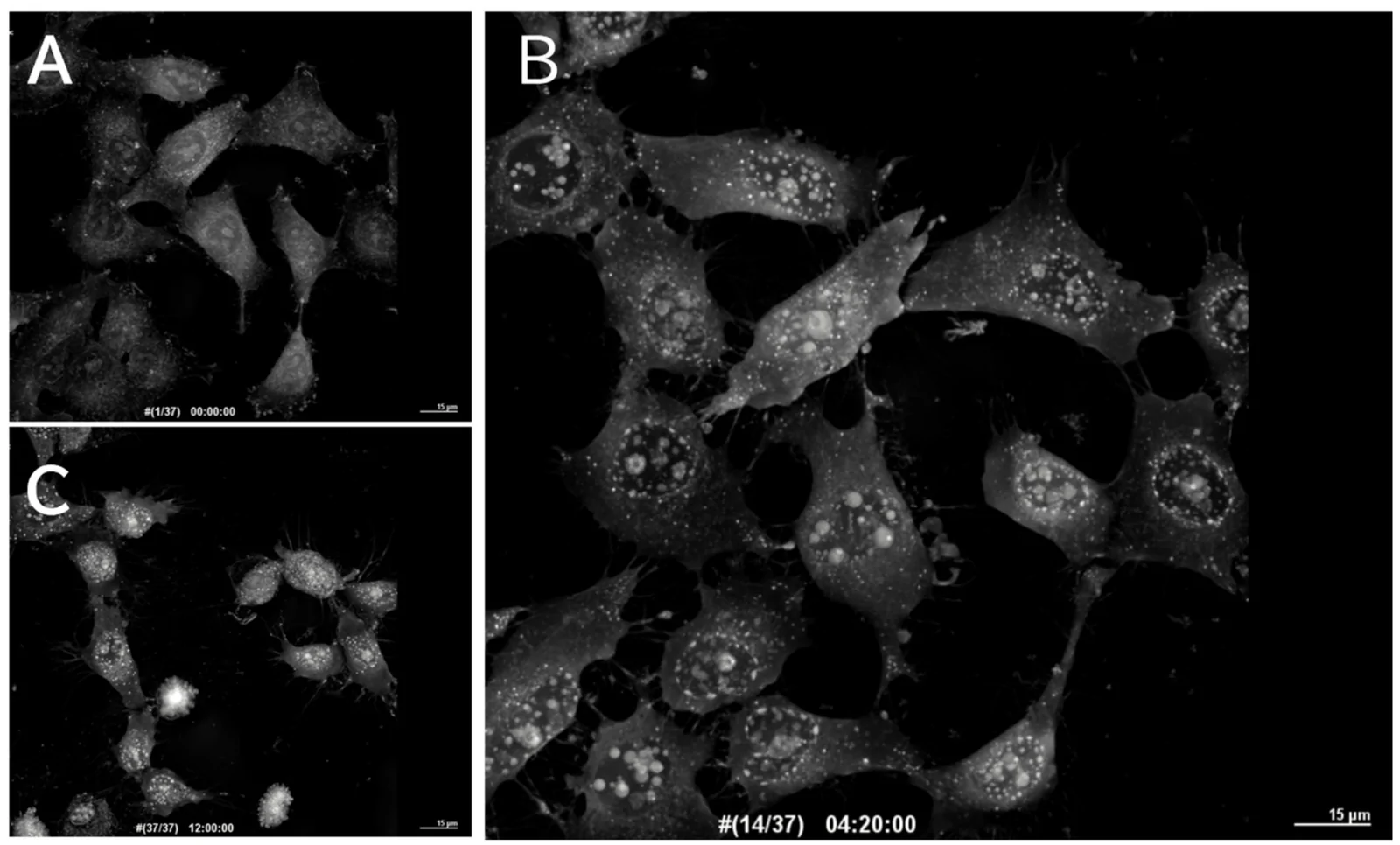
Holotomographic imaging of SOR-treated HeLa cells. Representative video frames captured at (**A**) 1 h, (**B**) 4 h, and (**C**) 12 h after treatment with 100 ng/mL SOR. Regions with a high refractive index (RI) are shown in white.

Next, the cellular responses after SOR treatment were analyzed by time-lapse holotomographic microscopy and fluorescence microscopy. Holotomographic microscopy is a label-free imaging technique that reconstructs the three-dimensional refractive index (RI) distribution of living cells from the phase delay of transmitted light acquired under multiple illumination angles, enabling quantitative visualization of cellular morphology and intracellular structures without exogenous labeling [15]. Because the cytological effects of SOR develop slowly, a relatively high concentration of SOR (100 ng/mL) was used to ensure detectable cellular changes within the 12 h observation period. At concentrations near the IC_50_, the effects required a longer time to become apparent. Holotomographic imaging acquired in a 12 h session showed that SOR-treated cells remained relatively unchanged during the early phase of incubation but underwent marked morphological changes after several hours (Figure 6, Supplemental Movie S1).

Cells gradually lost their spread morphology, accumulated highly refractive intracellular granules, and eventually became rounded and shrunken before cell death (Figure 6, Supplemental Movie S1). These observations indicate that SOR does not act primarily through acute membrane disruption. Rather, SOR appears to be internalized by cells, trafficked to the nucleus, and eventually accumulated in or around the nucleolus before inducing cell death.

Together with the DNase I-like activity of the N-terminal domain, these microscopic observations suggest that SOR functions as an intracellular genotoxin that induces nuclear damage by cleaving DNA, leading to apoptosis-like cell death.

**Figure 7:**
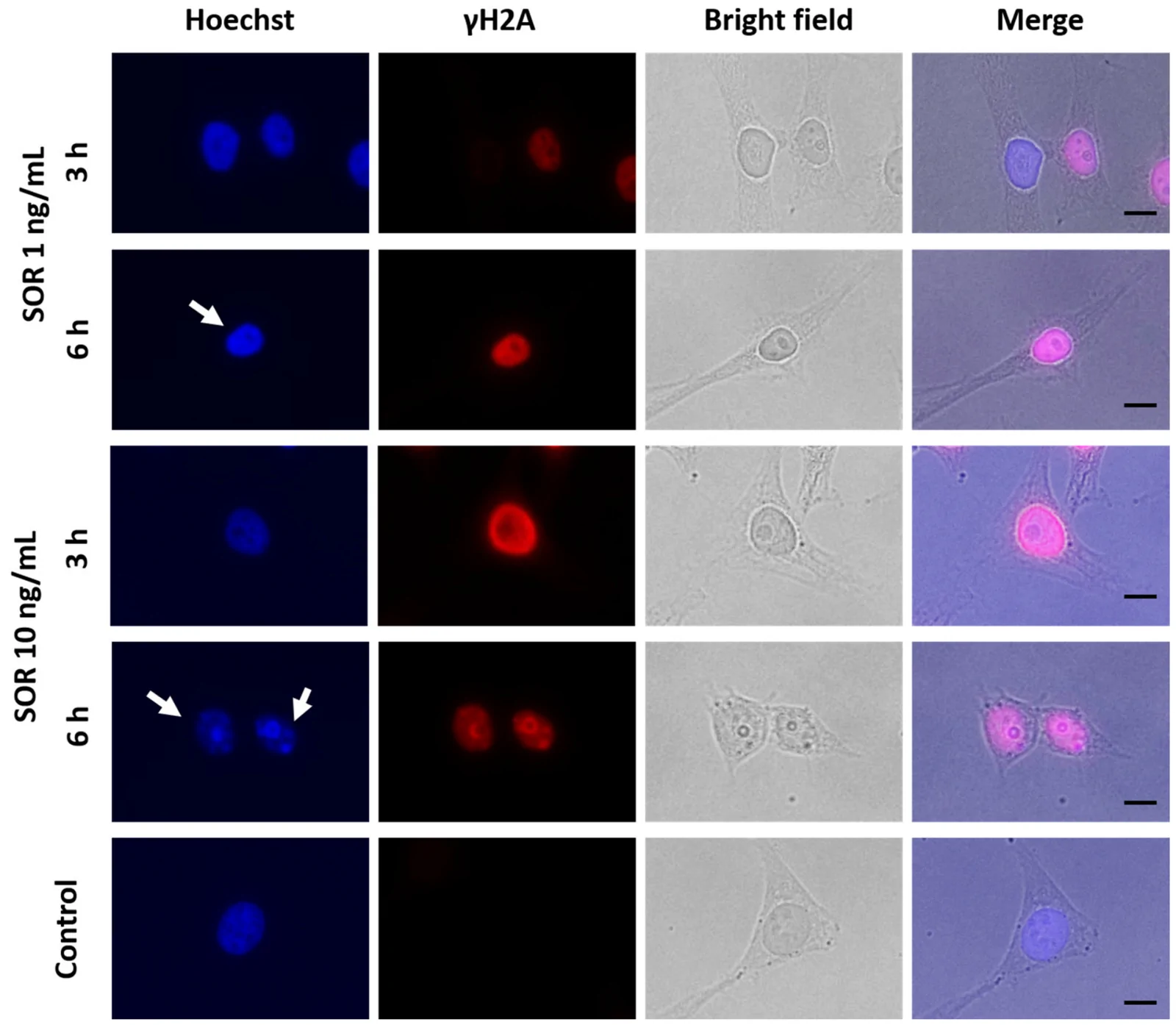
Fluorescence micrographs of HeLa cells treated with SOR. Fluorescent images of blue (Hoechst 33258), red (immunoreactivity for γH2AX), transmitted bright-field, and merged fluorescent images. Arrows indicate chromatin condensation. Scale bar = 10 μm.

To further investigate the nuclear function of SOR, SOR-treated HeLa cells were immunostained with an antibody against γH2AX (phospho-Ser139) [16]. Histone H2AX is rapidly phosphorylated at Ser139 in response to DNA double-strand breaks, and γH2AX is widely used as a highly sensitive marker of DNA damage. As expected, nuclei of SOR-treated cells were strongly positive for γH2AX at all concentrations tested (1, 10, and 100 ng/mL). At 6 h after treatment, γH2AX immunoreactivity was detected in a concentration-dependent manner. At the lowest SOR concentration tested (1 ng/mL), approximately half of the cells exhibited detectable nuclear γH2AX staining, whereas the remaining cells showed little or no immunoreactivity (Figure 7). The proportion of γH2AX-positive cells increased at higher SOR concentrations (Figure S11), indicating a concentration-dependent induction of the DNA damage response. Although these findings are consistent with DNA double-strand break formation, the present data do not directly demonstrate genomic DNA cleavage by nuclear-localized SOR. Notably, many γH2AX-positive cells treated with 1 ng/mL SOR retained apparently normal nuclear morphology, suggesting that H2AX phosphorylation occurred before the development of overt apoptotic changes [17] and that DNA damage is an early cellular response to SOR treatment.

## 3. Discussion

In the present study, we determined the complete coding sequence of *sor* by combining multiple PCR-based approaches guided by the N-terminal amino acid sequence and internal peptide sequences. Although conventional RACE alone was insufficient and several unexpected amplification events were encountered, these complementary strategies enabled reconstruction of the full-length coding sequence. The *sor* open reading frame comprises 2844 bp encoding a 947-amino-acid protein. Amplification of the complete coding region from both cDNA and genomic DNA yielded identical sequences, demonstrating that SOR is encoded as an intronless gene in the sponge genome [3,18]. These results also highlight the usefulness of combining complementary PCR strategies for determining the full-length sequences of large marine toxin proteins when conventional RACE approaches are unsuccessful.

The deduced sequence contains an additional 41-amino-acid peptide immediately upstream of the mature N-terminus identified by Edman degradation (Figure S4). This region is preceded by an in-frame TAA stop codon but does not exhibit the characteristics of a canonical signal peptide. It is therefore possible that this peptide represents an N-terminal propeptide that is removed during maturation of SOR.

Interestingly, although inverse PCR extended the genomic sequence upstream of the coding region, no unambiguous in-frame initiation codon corresponding to the *sor* open reading frame was identified. Furthermore, the inverse PCR product lacked the expected restriction enzyme junction, raising the possibility that the obtained fragment does not represent the authentic 5′ flanking region of the *sor* gene. Consequently, the translational initiation site and the mechanism underlying maturation of SOR remain unresolved and warrant further investigation.

### 3.1. Amino Acid Sequence of SOR

The amino acid sequence of SOR deduced from the gene *sor* was composed of a 41-amino-acid propeptide followed by a 947-amino-acid mature protein. Edman degradation determined the N-terminal 25-amino-acid sequence, which suggests that the propeptide was removed during protein maturation. However, the molecular size observed in MALDI-TOFMS (108 kDa) is considerably higher than the calculated size (103 kDa), which suggests posttranslational modifications, including glycosylation. Detailed structure analysis of native SOR is now underway in our laboratory.

The N-terminal region of SOR exhibited significant sequence similarity to DNase I and retained DNase activity, whereas the C-terminal region showed no detectable homology to any known protein family, making its function difficult to predict. Structural comparison revealed that five of the six catalytic residues essential for human DNase I activity (E39, E78, H134, D168, D212, and H252) [14,19] are conserved in SOR (E51, E94, H163, D193, D248, and H303).

Previous studies demonstrated that SOR activity is abolished by desalting using EDTA, consistent with the metal ion dependence of DNase I, in which divalent cations, particularly Mg^2+^, are essential for catalysis [3]. Together, these findings strongly support the conclusion that the N-terminal domain of SOR functions as a DNase I-like endonuclease.

Some bacterial toxins are known to utilize a DNase I-like catalytic module, most notably the cytolethal distending toxin (CDT) [13]. In CDT, the DNase I-like subunit CdtB is delivered into host cells by the receptor-binding subunits CdtA and CdtC, trafficked through the Golgi apparatus, and ultimately translocated into the nucleus, where it induces DNA damage, resulting in G2/M cell-cycle arrest and eventual cell death. Interestingly, expression of *Campylobacter jejuni* CdtB alone in COS-1 cells is sufficient to induce chromatin fragmentation, demonstrating that CdtB itself can translocate to the nucleus and exert its DNase activity independently of the other toxin subunits [20]. These observations support the idea that the catalytic subunit itself retains the intrinsic ability to access the nucleus. The present study suggests that SOR may employ a strategy related to that of CDT. However, unlike CdtB, which is a ~280-amino-acid catalytic subunit that functions together with separate delivery subunits, SOR is a single 947-amino-acid polypeptide. The lack of cytotoxicity of recombinant N-SOR suggests that the N-terminal DNase I-like domain alone is insufficient to reproduce the potent cytotoxicity of native SOR and that other regions of the protein are required for its full biological activity. However, the specific roles of these regions remain unclear, and the present data do not establish whether they are directly involved in cellular uptake, intracellular trafficking, nuclear translocation, protein stability, or other processes. Thus, SOR represents an unusual multidomain protein toxin in which a functional DNase I-like domain is incorporated into a single polypeptide, although the mechanisms by which the remaining regions contribute to its cytotoxicity remain to be determined.

### 3.2. Cellular Response After SOR Treatment

Holotomographic imaging revealed progressive morphological changes in HeLa cells following SOR treatment. Within several hours after exposure, cells exhibited an increase in highly refractive intracellular granules, followed by gradual cell shrinkage and eventual loss of adhesion without obvious plasma membrane rupture. These observations suggest that SOR does not primarily act as a membrane-disrupting toxin but instead induces intracellular stress responses after cellular uptake. Recent studies have demonstrated that 3D holotomography combined with deep-learning analysis can distinguish characteristic morphological features associated with different cell death pathways, including apoptosis, necroptosis, and necrosis [21]. Although the present morphological observations alone are insufficient to assign SOR-induced cell death to a specific pathway, the time-resolved 3D holotomographic data obtained in this study may provide additional mechanistic information in the future as well-characterized reference datasets and analytical frameworks for HT-based cell phenotyping become more widely established.

Fluorescence microscopy using FITC-labeled SOR further demonstrated that the toxin was internalized into HeLa cells through an energy-dependent process, transported to the perinuclear region, and subsequently translocated into the nucleus. Together with its DNase activity and the induction of γH2AX phosphorylation, these findings suggest that SOR cleaves nuclear DNA after reaching the nucleus.

The remarkable potency of SOR (IC_50_ = 0.5 pM in HeLa cells [10]), compared with that reported for CDT (~380 pM) [20], suggests that the activity of SOR cannot be explained solely by the catalytic properties of its DNase I-like domain. Consistent with this possibility, the recombinant N-terminal DNase fragment (N-SOR) did not exhibit detectable cytotoxicity. These observations suggest that regions outside the N-terminal DNase I-like domain are important for the potent cytotoxicity of SOR, potentially by contributing to cellular uptake, intracellular trafficking, protein stability, or other biological activities. However, the specific contributions of these regions remain to be experimentally determined.

## 4. Conclusions

In conclusion, the present study identifies SOR as a previously undescribed marine sponge toxin that combines a functional DNase I-like domain with an efficient intracellular delivery module in a single polypeptide. Although conceptually analogous to bacterial A–B toxins, SOR represents the first example of such a proteinaceous toxin from marine sponges. Our findings establish a new class of marine genotoxins and provide a foundation for elucidating novel mechanisms of intracellular trafficking and nuclear targeting that may inspire future protein-based drug delivery technologies.

## 5. Materials and Methods

### 5.1. RNA Extraction, cDNA Synthesis, and Genomic DNA Preparation

A specimen of the marine sponge *Spongosorites* sp., collected from Iriomote Island, Okinawa, Japan, in 2002, was used for cDNA synthesis and genomic DNA extraction. Immediately after collection, sponge tissues were immersed in RNAlater (QIAGEN, Hilden, Germany), stored at −20 °C for several days, and subsequently transferred to −80 °C until use.

Total RNA was extracted using the RNeasy Mini Kit (QIAGEN) according to the manufacturer’s instructions. The concentration and purity of the isolated nucleic acids were determined using a NanoDrop spectrophotometer (Thermo Fisher Scientific, Waltham, MA, USA), and RNA integrity was assessed by electrophoresis on a 0.8% agarose gel. Unless otherwise stated, all PCR amplifications were performed by nested PCR using Phusion High-Fidelity DNA Polymerase (New England Biolabs, Ipswich, MA, USA). PCR products were purified from agarose gels using the AxyPrep DNA Gel Extraction Kit (Axygen, Union City, CA, USA) and subjected to DNA sequencing. Primer sequences are listed in Table S1.

First-strand cDNA synthesized using the SMARTer RACE cDNA Amplification Kit (Takara Bio, Shiga, Japan) was used as the template for PCR amplification. Degenerate primers (SOR1Fw, SOR1Rev, SOR2Fw, and SOR4Rev; Table S2) were designed based on the N-terminal amino acid sequence and internal peptide sequences of SOR determined by Edman degradation. Nested PCR was performed using KOD Dash DNA polymerase (Toyobo, Osaka, Japan) under the conditions summarized in Table S2. The first-round PCR products served as templates for the second-round PCR. Amplified fragments of approximately 300 bp were separated by agarose gel electrophoresis, purified using the AxyPrep DNA Gel Extraction Kit (Axygen), and subjected to DNA sequencing.

### 5.2. 3′-RACE Analysis of the SOR Transcript

To obtain the 3′-terminal region of the sor transcript, gene-specific primers (SOR5Fw1 and SOR5Fw2) were designed based on the 884 bp sequence obtained from the mis-priming PCR experiment. Nested 3′-RACE was performed using 3′-RACE-ready cDNA as a template. The first PCR was carried out using SOR5Fw1 and the universal primer mix (UPM), whereas the second PCR was performed using the first-round PCR product as a template with SOR5Fw2 and the nested universal primer (NUP). Phusion High-Fidelity DNA Polymerase (New England Biolabs) was used for all PCR reactions.

The second-round PCR products were separated by agarose gel electrophoresis, and a DNA fragment of approximately 2.5 kb was excised and purified using the AxyPrep DNA Gel Extraction Kit. Cloning and sequencing of the amplified products yielded four independent clones containing a poly(A) tail at the 3′ end.

#### 5.2.1. Inverse PCR

Genomic DNA was digested with the restriction enzyme Pst 1. The digested DNA was purified using the AxyPrep PCR Cleanup Kit (Axygen), self-ligated using a commercial ligation mixture at 4 °C overnight and used as the template for inverse PCR. Gene-specific primers SOR3Fw2, SOR-inv1, SOR-inv2, and SOR4Fw2 (Table S2) were designed based on the sequence obtained by the initial first-strand PCR. Nested inverse PCR was performed using Phusion High-Fidelity DNA Polymerase (New England Biolabs), with the first-round PCR product serving as the template for the second-round reaction. An amplified fragment of approximately 1.5 kb was separated by agarose gel electrophoresis, purified using the AxyPrep DNA Gel Extraction Kit (Axygen), and subjected to sequence analysis.

#### 5.2.2. Full-Length PCR

Both 3′-RACE-ready cDNA and genomic DNA were used as templates. Gene-specific primers (SOR5′-5, SOR3′-2, SOR5′-4, and SOR3′-1; Table S1) were designed from the inverse PCR and 3′-RACE sequences to amplify the complete sor coding region.

### 5.3. Cloning and Recombinant Expression of Full-Length SOR

The full-length *sor* coding sequence was amplified from the 5′-RACE-ready cDNA using Phusion High-Fidelity DNA Polymerase (New England Biolabs). PCR primers containing SalI and EcoRI restriction sites (Table S1) were used for cloning into the pEcoli-Nterm-6×HN expression vector (Clontech, Mountain View, CA, USA). The resulting plasmid was propagated in *E. coli* NEB 10-beta and transformed into SHuffle T7 Express lysY cells (New England Biolabs) for protein expression. Cultures were grown in LB medium containing ampicillin (100 μg/mL) at 30 °C until an OD600 of 0.4–0.8 was reached, and protein expression was induced with 0.4 mM IPTG for 4 h. Cells were harvested, disrupted by sonication in 50 mM Tris-HCl (pH 7.4) containing 150 mM NaCl, and separated into soluble and insoluble fractions by centrifugation. Recombinant SOR expression was analyzed by SDS-PAGE.

### 5.4. Cloning of the Euryhalocaulis caribicus SOR Homolog

*E. caribicus* was obtained from RIKEB RBC (JCM18163) and cultured in cMB medium at 30 °C for 4 days with shaking at 200 rpm. Genomic DNA was extracted using the DNeasy Blood & Tissue Kit (QIAGEN). Gene-specific primers (Table S1) were designed based on the genomic sequence of *E. caribicus* (GenBank accession no. ASJA01000002), and the SOR homolog (ECP) was amplified by nested PCR using Phusion High-Fidelity DNA Polymerase. PCR products of approximately 3 kb were purified from agarose gels using the AxyPrep DNA Gel Extraction Kit (Axygen).

### 5.5. AlphaFold 3 Modeling and Foldseek Analysis

The three-dimensional structure of full-length SOR was predicted using AlphaFold 3 [22]. Based on the predicted structure, SOR was divided into four putative structural domains. Each domain was individually subjected to structural similarity searches using Foldseek [23] against the AFDB50 database [24]. Structural similarities were evaluated based on Foldseek E-values, sequence identities, and alignment coverage. Representative structural matches were selected primarily based on E-value and query coverage.

### 5.6. Recombinant Expression of N-SOR

The N-terminal region of sor (residues 1–310) was synthesized commercially and cloned into the pET-22b(+) vector using SalI and HindIII restriction sites. The recombinant plasmid was transformed into SHuffle T7 Express lysY cells for protein expression. Cultures were grown in LB medium and induced with 0.4 mM IPTG at 30 °C for 4 h.

Because the recombinant protein was produced predominantly as inclusion bodies, the insoluble fraction was isolated, washed with octylphenol ethoxylate, solubilized in 8 M urea, and purified by Ni^2+^-affinity chromatography under denaturing conditions.

The purified protein sample was transferred to cellulose dialysis tubing (UC36-32-100; molecular weight cutoff, 14 kDa; pore size, 50 Å; Sanko Junyaku, Tokyo, Japan) and refolded by dialysis against 50 mM Tris-HCl (pH 9.0) containing 0.1 M NaCl for 5 days at 20 °C. The dialysis buffer was replaced once daily. After dialysis, the sample was recovered from the tubing and centrifuged at 8000 rpm for 10 min. The resulting supernatant was collected and used as the refolded N-SOR preparation for subsequent experiments.

DNase activity was evaluated by incubating recombinant N-SOR with a PCR product using pBS SK(+) plasmid DNA as a template in Tris-HCl buffer (pH 8.3) in the presence or absence of EDTA. DNA cleavage products were analyzed by agarose gel electrophoresis and visualized by ethidium bromide staining.

### 5.7. Cell Culture and Cytotoxicity Assay

Cytotoxicity of N-SOR was evaluated using murine Ba/F3 cells [25]. Ba/F3 cells were adjusted to 2.5 × 10^3^ cells/mL and seeded into 96-well plates at 90 µL per well. A sample of N-SOR (10 µL) was added to each well at a concentration of 330 ng/mL. After incubation for 36 h in a CO_2_ incubator, cell numbers were estimated by microscopic observation. Cell viability was expressed relative to that of the untreated control after 36 h, which was defined as 100%.

HeLa cells were used to evaluate native SOR. The cells (RBRC-RCB0007; Riken, Saitama, Japan) were maintained in Dulbecco’s modified Eagle’s medium (DMEM) supplemented with 10% fetal bovine serum and penicillin–streptomycin at 37 °C under 5% CO_2_. For cytotoxicity assays, cells were seeded in 96-well plates at 5.0 × 10^3^ cells per well, cultured overnight, and treated with the indicated samples for 48 h. Cell viability was determined using a WST-8 assay kit (Dojindo Laboratories, Kumamoto, Japan). After incubation with WST-8 reagent for 2 h, absorbance at 450 nm was measured.

### 5.8. Holotomographic Imaging

Holotomographic imaging of HeLa cells was performed using a Tomocube HT-X1 system (Tomocube Inc., Daejeon, Republic of Korea). HeLa cells were seeded in 24-well plates at 2.0 × 10^4^ cells per well and cultured overnight. The medium was then replaced with Opti-MEM, and the cells were treated with SOR at 100 ng/mL to monitor morphological changes over 12 h. Images were acquired every 20 min throughout the observation period.

### 5.9. FITC Labeling of SOR

SOR in 0.1 M carbonate buffer (pH 9.0; 2 mg/mL) was mixed with 50–100 μL of freshly prepared FITC-I solution in anhydrous DMSO (1 mg/mL) per milliliter of protein solution. The mixture was incubated overnight at 4 °C in the dark. Free FITC-I was removed by ultrafiltration, and the buffer was exchanged for PBS. The labeled protein was designated FITC-SOR.

### 5.10. Live-Cell Fluorescence Microscopy

HeLa cells were seeded in 96-well glass-bottom plates at 5.0 × 10^3^ cells per well and cultured overnight. The medium was replaced with Opti-MEM™ I Reduced Serum Medium, and FITC-SOR was added at 10 μg/mL. After incubation for the indicated periods, cells were washed with PBS, stained with Hoechst 33258 (5 μg/mL) for 15 min, and observed using a BZ-X800 fluorescence microscope (Keyence, Osaka, Japan).

### 5.11. Immunofluorescence Staining

HeLa cells were seeded on glass coverslips in 24-well plates at 2.0 × 10^4^ cells per well and cultured overnight. After treatment in Opti-MEM, cells were fixed with 4% paraformaldehyde for 15 min, permeabilized with 0.1% Triton X-100 for 5 min, and blocked with 5% bovine serum albumin for 30 min.

Cells were incubated with rabbit monoclonal anti-γH2AX (phospho-Ser139) antibody [EP854(2)Y] (1:250; ab81299, Abcam, Cambridge, UK) for 1 h, followed by Alexa Fluor 594-conjugated goat anti-rabbit IgG (1:500; ab150092, Abcam) for 1 h. Coverslips were mounted with ProLong™ Diamond Antifade Mountant (Thermo Fisher Scientific, Waltham, MA, USA) with DAPI and imaged using a BZ-X800 fluorescence microscope (Keyence Corporation, Osaka, Japan).

### 5.12. Nucleolar Staining

HeLa cells were seeded in 96-well glass-bottom plates at 5.0 × 10^3^ cells per well and treated in Opti-MEM. Cells were fixed with 4% paraformaldehyde, permeabilized with 0.1% Triton X-100, and stained with Nucleolus Bright Red (1:1000; Dojindo Laboratories, Kumamoto, Japan) for 15 min. Images were acquired using a BZ-X800 fluorescence microscope.

## Data Availability

The original contributions presented in this study are included in the article/Supplementary Material. Further inquiries can be directed to the corresponding authors.

## Supplementary Materials

The following supporting information can be downloaded at: https://www.mdpi.com/article/10.3390/toxins18100434/s1, Table S1: Experimentally determined N-terminal and internal peptide sequences of SOR, Table S2: Oligonucleotide primers used for SOR cloning and RACE experiments, Figure S1. Nucleotide sequence of the first-strand PCR product and the deduced amino acid sequence. Figure S2. Nucleotide sequence of the mis-primed PCR product and the deduced amino acid sequence. Figure S3. Nucleotide sequence of the 3′ RACE product and the deduced amino acid sequence of the coding region. Figure S4. Inverse-PCR strategy. Figure S5. Nucleotide sequence and deduced amino acid sequence of full-length natural SOR. Figure S6. Alphafold-3 followed by Foldseek analysis of SOR and its domains. Figure S7. Expression of recombinant SOR and ECP in *Escherichia coli*. Figure S8. Expression and purification of N-SOR. Figure S9. Bioactivity of SOR and FITC-SOR used in this study. Figure S10. Fluorescent micrographs of HeLa cells treated with FITC-SOR. Figure S11. Proportions of immunostained cells at 2 and 6 h after SOR treatment. Movie S1: Holotomographic imaging of SOR-treated HeLa cells.
