# Supplementary material for: Molecular Characterization of Soritesidine Reveals a Novel Marine Sponge DNase I-like Toxin
Source: Toxins (Basel). 2026 Oct 9;18(10):434. doi: 10.3390/toxins18100434 (PMC13709786; doi:10.3390/toxins18100434)

Supplementary Materials: Molecular Characterization of Soritesidine Reveals a Novel Marine Sponge DNase I-Like Toxin

Hiromi Watari, Koji Nakano, Masaya Kitano, Hiromu Kageyama, Yoshikazu Tanaka, Yuri Ishibashi, and Ryuichi Sakai

Contents

Supplementary tables

Table S1. Experimentally determined N-terminal and internal peptide sequences of SOR.

Table S2. Oligonucleotide primers used for SOR cloning and RACE experiments.

Supplementary figures

Figure S1. Nucleotide sequence of the first-strand PCR product and the deduced amino acid sequence.

Figure S2. Nucleotide sequence of the mis-primed PCR product and the deduced amino acid sequence.

Figure S3. Nucleotide sequence of the 3′ RACE product and the deduced amino acid sequence of the coding region.

Figure S4. Inverse-PCR strategy.

Figure S5. Nucleotide sequence and deduced amino acid sequence of full-length natural SOR.

Figure S6. Alphafold-3 followed by Foldseek analysis of SOR and its domains.

Figure S7. Expression of recombinant SOR and ECP in *Escherichia coli*.

Figure S8. Expression and purification of N-SOR.

Figure S9. Bioactivity of SOR and FITC-SOR used in this study.

Figure S10. Fluorescent micrographs of HeLa cells treated with FITC-SOR.

Figure S11. Proportions of immunostained cells at 2 and 6 h after SOR treatment.

Supplementary Movie

Movie S1: Holotomographic imaging of SOR-treated HeLa cells.

Supplementary tables

Table S1. Experimentally determined N-terminal and internal peptide sequences of SOR. Red letters are amino acid sequences matched with the full-length amino acid sequence of SOR (see Figure S5).

No.	Assignment	Amino acid sequence
1	N-terminal sequence	KLGDQRQIDIASWNTFDGGVXKAN
2	Internal peptides	XGH
3		TGAXXXE
4		SGXST
5	Internal 346–357	(Q/G)AGFVPNXTXDXT

No.	Assignment	Amino acid sequence
6	Internal 509–512	NFDY
7		LALEVPLRTVNXT
8		GDGENXNDNXDXYD
9		ASTGSTIPXGXXT
10	Internal 656–664	ESAAETEN(E/G)
11		ASAAPTNN(N/A)XTSLSSGXD(E/G)
12		XLD(D/E)ETLE
13	Internal 522–528	PLDVRGTY(D/E)XXXV
14		HXQDRIQPAXPPXH
15	Internal 101–130	ANTGIGNVVIERTDNPNTVPYIPA(A/G)GXNNNHXH
16	Internal 185–197	FAVITLGDLNADGXH
17		LPG

Sequence notation: X indicates an undetermined residue; alternatives in parentheses indicate ambiguous assignments reported in the source file.

Table S2. Oligonucleotide primers used for SOR cloning and RACE experiments.

No.	Primer	Direction	Sequence (5′→3′)	Length (nt)
1	SOR1Fw	Forward	CARMGNCARATHGAYATHGCN	21
2	SOR1Rev	Reverse	ATNGYNSWNCCNGTNSWNGC	20
3	SOR2Fw	Forward	TGGAAYACNTTYGAYTTYGG	20
4	SOR4Rev	Reverse	CCNGCNGCNGGDATRTANGG	20
5	SOR3Fw2	Forward	CAAATGTCACAAATCCATGTAATCCTGC	28
6	SOR4Fw1	Forward	ATGATCCTACCATACTGCAAGC	22
7	SOR4Fw2	Forward	GATACTGGCATTGTAGGCTCTGG	23
8	SOR5Fw1	Forward	GGCACTCTCTGATCATAGACTG	22
9	SOR5Fw2	Forward	CAGCTGATGAGGTATACGTCAAAGG	25
10	SOR-inv1	Reverse	AATCTCTTGAACCAAAATGACATCATGC	28
11	SOR-inv2	Reverse	CTTACATACACCGCCAAACTTGC	23
12	SOR3'-1	Reverse	GTGCTGGTTTTAGATGTGTGGCTTGTGC	28
13	SOR3'-2	Reverse	TCTGGACATATCAAGGCCCTGCAGGTGG	28
14	SOR5'-4	Reverse	CCATTAAACTTGAAAACATGACAGGGCC	28
15	SOR5'-5	Reverse	TGATCTTACAGTACAACCAGCAGCACTG	28
16	UPM (Long)	Forward	CTAATACGACTCACTATAGGGCAAGCAGTGGTATCAACGCAGAGT	45
17	UPM (Short)	Forward	CTAATACGACTCACTATAGGGC	22
18	NUP	Forward	AAGCAGTGGTATCAACGCAGAGT	23
19	rSORFw	Forward	GTCGACAAGCTTGGAGATCAGCGACAG	27
20	rSORRev	Reverse	GAATTCCTAACAGCCAGTACCTGGAAG	27

Figure S1. Nucleotide sequence of the first-strand PCR product and the deduced amino acid sequence.

Nucleotide sequence (345 nt; 5′→3′)

```
  1  TGGAATACTT TTGACTTTGG TGGCAAGTTT GGCGGTGTAT GTAAGGCAAA TGATCCTACC     60
 61  ATACTGCAAG CAATGACTGA TGTAATATCT CAGCATGATG TCATTTTGGT TCAAGAGATT    120
121  GCAAATGTCA CAAATCCATG TAATCCTGCA AACATCTTTT CAGGCTTTAA TAATTTATGT    180
181  TCAAATAACT ATCTTGGCGG TCTGGGATAT CTCTGCCAAG ATACTGGCAT TGTAGGCTCT    240
241  GGTGAGGGCT ATGGTATTAT CTACAAAAAC AACACCGGTA TTGGTAATGT AGTAATTGAG    300
301  CGTACAGATA ACCCAAATAC AGTTCCATAT ATACCAGCAG CAGGG                    345
```

Inferred amino-acid sequence (115 aa)

```
 1  WNTFDFGGKF GGVCKANDPT ILQAMTDVIS QHDVILVQEI ANVTNPCNPA NIFSGFNNLC SNNYLGGLGY     70
71  LCQDTGIVGS GEGYGIIYKN NTGIGNVVIE RTDNPNTVPY IPAAG                    115
```

Figure S2. Nucleotide sequence of the mis-primed PCR product and the deduced amino acid sequence.

A. Nucleotide sequence (884 nt; 5′→3′)

```
  1  GATACTGGCA TTGTAGGCTC TGGTGAGGGC TATGGTATTA TCTACAAAAA CAACACCGGT   60
 61  ATTGGTAATG TAGTAATTGA GCGTACAGAT AACCCAAATA CAGTTCCATA CATTCCTGCA  120
121  GGTGGTGGCA ACAACCAAAT GAAGAGGCCA CCAATGAAGG CAACCCTTGA CTTTAATGCA  180
181  GGTGCATTCA ACGTAGTAGT ATATAATGCA CACACAAGCC CAAGTGGCGG TGTTCCCAAA  240
241  GAGATAAAGC TACTCAGTGA TAGAATATCG CCTGCTCACA ATCCGCTAGA TAAAAACGCA  300
301  GTAATTACAC TAGGTGATCT TAATGCCGAC GGCAAAAGAA GACTGCCTAA TGGACAGCTA  360
361  GACCATGGAA CAGACTATTA CAGAGGTGGC TTTGCAGCTC ACCCAAATGA CTTTCCTGTT  420
421  AGTGGATGGC AGCAGACAGT TACTGATACT GATGTTACAA ACTTTGCACA GACACTTCGT  480
481  GCATATGATA GAATGATACT TAGCAATGCA ACACATAACT CTTATGATGA GTATGGAATA  540
541  ATAGGTGGCA TTCCAAACCC ACTTCCATAT CCGGGAGGAG TTTATCCTGC TGGAACTTTG  600
601  TTTAAAAATG TACATACTGG CGGTGGAAAT ACAGGAAAGG CACTCTCTGA TCATAGACTG  660
661  ATATGGTCAA AGCTTAACTA TGGTGCAATA GAGACTACCG ATAATAACGG CAATACAAAG  720
721  TCCAATTTTG TTGAATTTAC AAACCCACCA CAATGTACTG CACCAGATAC CGTATATGCA  780
781  AAGGGTGCAG ACTTTGTACC AAATAGAACA GTTGATATTT ACATCACAAA GCAGCCAGAC  840
841  AATCAAAACT TTATCCCACT GCCAGAGCCT ACAATGCCAG TATC                   884
```

B. DNA-supported deduced amino acid sequence (288 aa). Residues matching the Edman-derived peptide sequence are shown in bold.

1 DTGIVGSGEG YGIIYKN**NTG IGNVVIERTD NPNTVPYIPA G**G**GNN**QMKRP PMKATLDFNA GAFNVVVYNA 70

71 HTSPSGGVPK EIKLLSDRIS PAHNPLDKN**A VITLGDLNAD G**KRRLPNGQL DHGTDYYRGG FAAHPNDFPV 140

141 SGWQQTVTDT DVTNFAQTLR AYDRMILSNA THNSYDEYGI IGGIPNPLPY PGGVYPAGTL FKNVHTGGGN 210

211 TGKALSDHRL IWSKLNYGAI ETTDNNGNTK SNFVEFTNPP QCTAPDTVYA K**GADFVPN**R**T** V**D**IYITKQPD 280

281 NQNFIPLPEP TMPVS 295

C. Mechanism of coincidence priming

```
SOR4Fw2           GAT ACT GGC ATT GTA GGC TCT GG
Inverted SOR4FW2  GG TCT CGG ATG TTA CGG TCA TAG
3′ sequence       CC AGA GCC TAC AAT GCC AGT ATC
```

Figure S3. Nucleotide sequence of the 3′ RACE product and the deduced amino acid sequence of the coding region.

A. 3′ RACE product (2511 nt; 5′→3′)

```
   1  GGCACTCTCT GATCATAGAC TGATATGGTC AAAGCTTAAC TATGGTGCAA TAGAGACTAC     60
  61  CGATAATAAC GGCAATACAA AGTCCAATTT TGTTGAATTT ACAAACCCAC CACAATGTAC    120
 121  TGCACCAGAT ACCGTATATG CAAAGGGTGC AGACTTTGTA CCAAATAGAA CAGTTGATAT    180
 181  TTACATCACA AAGCAGCCAG ACAATCAAAA CTTTATCCCA CTGCCACAGC CACAAAACCC    240
 241  AACAAACCCT GGGCAGTTCA AGCAAGTTGG CGAATCATAC CAGCTAACAG ATGTAAGGAC    300
 301  TACAGGCAAA AGTACGCACA ACATTGATGC AAACGGCAAC ATTGCAAATA CTGCTGTATG    360
 361  GGTACAGCCA ACACCAAACC AGCAGTACTA CCTCATAGTT GATGTGGACC GTGATGGATA    420
 421  CTTTACAAAC AGCATAGATA TAGTTGATGC ATTTAACACA CAAGGCTTTG ATGTAGACCC    480
 481  ATGCCTTCGA AAGAGACTAG CAGCATCACT TTCTGATGGA ATACCAGCAG GCGATGGCAC    540
 541  ACGTGACTTT GTAAAACCTC CCAAAATATT TATTTCCGGT GACTGGTTCC CACAGGGTTC    600
 601  TGCACGTATC TACATTATCA TATATGACAA GAACTTTGAT TATAACGGCA ATGACTCCAA    660
 661  AGTATTAACT GACGTCCGTG GAACTTATGA CACAATTACA GTGGGTGAGA ACCACAAGTT    720
 721  TACAGGACTA GAATGGTCCT CACCACATGT TGGTAACTAT AACATAATAT TTGATGCAAA    780
 781  CTCTGATGGA GTATACAAAA AATCCGATGG TGACATTGTA GATCACATAA ACGAGATAGG    840
 841  CTTTGCAATG CTCGAATCGT ATCCACATCA CAGCCATATT GTAGATTTAG ATGACAATGC    900
 901  AAACCAGCGA GAAGACTTTA ACAGTGGAAT TGCAAAAAAC GTCTACGTTC TTGCAACTAA    960
 961  TCTTCCAAAC CCAAGCACAA TAGTTGACGT TTATGTAATT AGTGAGAAAC TCTTAAAGCT   1020
1021  AAACAATCCT GGATGGACCA CATGGCAGCA ATCCTCTAAT GTGCTACTAA AGGAGAGTGC   1080
1081  AGCACCAACT AACAATGGAG ACAGGCTACA CCCTACTTGG GTAACTCCAC AAAAGACAGT   1140
1141  ATTTGGTTCA GTATGGCATA TGCCCGGTGA TACATTTGAT CCACCATACA TTAATTATTA   1200
1201  CGGCAAAAAA TTCACTATAG TAATTGATGT GAATCGTGAT GGTAAGTATG ATCCTAGTAT   1260
1261  AGATATGGTA GATACTCACG ATATAGGAGA CATGACAGAC TGGTTTATCA CACAGGGTCA   1320
1321  CCATGACCTA AGCCCAACTA GTGGAAACGG ACAGCCTGCC GTATCTGAAT ACAAAGAGTA   1380
1381  TCTCAACTCA AAGCTAGACT TGGAGAACCC GCTTGATAAC ACCAACAACT ATGATGCTGC   1440
1441  AACTGAGGCA GCTTCTGCAC AATATCTATG CCGTACCAAT CTTCCAATCA ATCTATTTGA   1500
1501  GAATGTCATA GAGCTGGGAG CTCAGACAGG CTTTATCATG TTTAACCCTG ATGAGTATTA   1560
1561  TGCAGGCCGT GATCTAAATT CTGGACGCCA CATTTATGAT GCTGTAGATT TTGATGGCTT   1620
1621  GGAGATAAAT GACGGATACA CATCAGTAAT CTCTGCAAAA GAGATGACCC TAAACAGATT   1680
1681  AGGTGTTGGC CAAAACTCCA ATCTGCTTAC CTGCGGAGCT GACACAGTAA CGATAGATGG   1740
1741  CATGTCAACA CAAAAAGATT CAACTACAAA TATTGTTGCA AAGACCATAT CTCACAAAAG   1800
1801  TAACATCTCA CTAGAGGCAG ACACTTGTAC ACATTATACT CACAATCTCT ACTTTGAGTC   1860
1861  AGACACCAAC ATCTTAGTTA AGGCCATTGA GAGTCCATTT GCATGGATAA TTGACGTAGG   1920
1921  TGACAGAATA CTTCCAGGTA CTGGCTGTTA GATGAATCGT GTGTGTGGGT TCTTCTAATA   1980
1981  TGATTGCACA AGCCACACAT CTAAAACCAG CACAATGCAC ACACACCTTC TGCTGTTTTA   2040
2041  TAACTTTAAT ACATTATGAC ATTTTACTCT AAATTGCAAT AATAGTTAAT CAGTGTATAT   2100
2101  ACTCAAAAAC AAAAAACTAT CCCTCATACC ATTGAGTGCT ATGATATTAT TTTTGGGAAT   2160
2161  AATAATAATA ATAATGGGGG GGGGCATCTT CGCAGTCCTT TTCTCAAACT GATATATAGA   2220
2221  TAAACTAAAT GACAATTCAC AAGATCTGGG TAATCTATAG ATTACTTAAC ATGATGTTAT   2280
2281  CATGCTATCC TTTCTATGGG GGCCACCTGC AGGGCCTTGA TATGTCCAGA TAAAAAACAG   2340
2341  GTCAAATTAC GTCCCACCTT TTGTACAGAT CTCCTACTAT CGAATATCTA AAATAACGGC   2400
2401  TAAAATAGCC AAACGCTAGG AAGGTTATCT AATGAGCTTA AAAAAAAAAC AAGAAACAAA   2460
2461  AAAAAAAAAA AAAAAAAAAA AAAAAAGTAC TCTGCGTTGA TACCACTGCT T              2511
```

B. Inferred coding-region amino-acid sequence (649 aa; translation begins at nucleotide 2)

```
  1  ALSDHRLIWS KLNYGAIETT DNNGNTKSNF VEFTNPPQCT APDTVYAKGA DFVPNRTVDI YITKQPDNQN     70
 71  FIPLPQPQNP TNPGQFKQVG ESYQLTDVRT TGKSTHNIDA NGNIANTAVW VQPTPNQQYY LIVDVDRDGY    140
141  FTNSIDIVDA FNTQGFDVDP CLRKRLAASL SDGIPAGDGT RDFVKPPKIF ISGDWFPQGS ARIYIIIYDK    210
211  NFDYNGNDSK VLTDVRGTYD TITVGENHKF TGLEWSSPHV GNYNIIFDAN SDGVYKKSDG DIVDHINEIG    280
281  FAMLESYPHH SHIVDLDDNA NQREDFNSGI AKNVYVLATN LPNPSTIVDV YVISEKLLKL NNPGWTTWQQ    350
351  SSNVLLKESA APTNNGDRLH PTWVTPQKTV FGSVWHMPGD TFDPPYINYY GKKFTIVIDV NRDGKYDPSI    420
421  DMVDTHDIGD MTDWFITQGH HDLSPTSGNG QPAVSEYKEY LNSKLDLENP LDNTNNYDAA TEAASAQYLC    490
491  RTNLPINLFE NVIELGAQTG FIMFNPDEYY AGRDLNSGRH IYDAVDFDGL EINDGYTSVI SAKEMTLNRL    560
561  GVGQNSNLLT CGADTVTIDG MSTQKDSTTN IVAKTISHKS NISLEADTCT HYTHNLYFES DTNILVKAIE    630
631  SPFAWIIDVG DRILPGTGC                                                 649
```

Figure S4. Inverse-PCR strategy.

A. Schematic representation of the inverse-PCR strategy used to recover sequence upstream of the known SOR fragment.

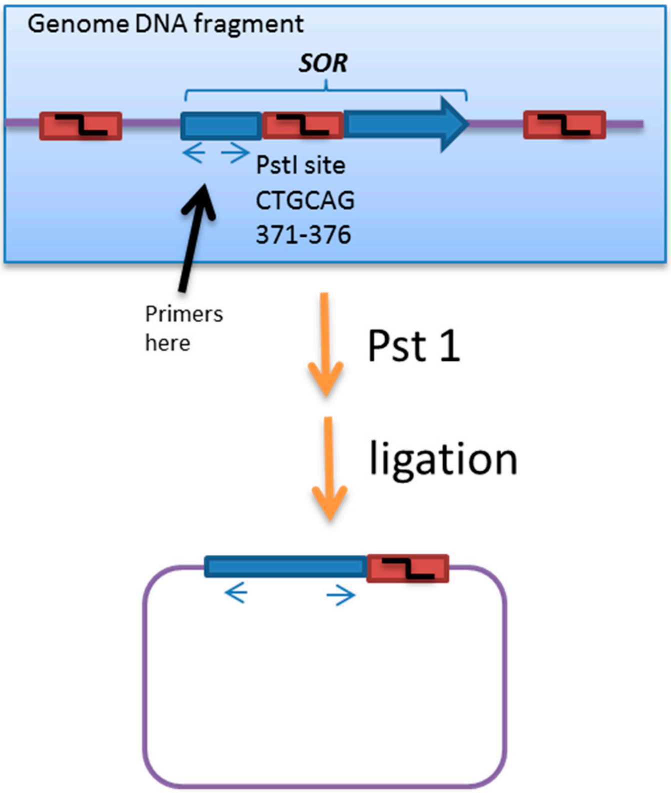

B. Nucleotide sequence obtained by inverse PCR. Primer-binding sites are highlighted in blue, the *PstI* restriction site is highlighted in yellow at the unmatched junction, the N-terminal sequence is shown in red, and the 41-amino-acid propeptide is shown in green. Amino acid residue numbers are shown on the right.

SORFw2⇒

	GAT	ACT	GGC	ATT	GTA	GGC	TCT	GGT	GAG
86	D	T	G	I	V	G	S	G	E
	GGC	TAT	GGT	ATT	ATC	TAC	AAA	AAC	AAC

95	G	Y	G	I	I	Y	K	N	N
	ACC	GGT	ATT	GGT	AAT	GTA	GTA	ATT	GAG
104	T	G	I	G	N	V	V	I	E
	CGT	ACA	GAT	AAC	CCA	AAT	ACA	GTT	CCA
113	R	T	D	N	P	N	T	V	P
	TAC	A							
122	Y								

GGCAATGACTATAGATAGTACAGGCAAGGTAGGAATTGGAACTACTAGTCCAAATGCAAAATTACATGTACAAGGATCATTTA
GAGTCCAAGGCTATAACATCTTAACTGACCCTGATGAACAATACAACAGTCATTTTCCTCATTCAAGTAATAATGTCTACATCT
CCGGTGATAAAACATATCTTAGAAGTGGCAAACCAAACGGTTACTCCACTACAATGACTGTTGATAACATCAATGGTAATGTA
GGCATTGGACTAAAAGAGCCCAAAGTAAAACTTCATATGTTTGGTGATGCCTTTTTGCAAACAGGCTCTTCTGGCGATTCAAGA
AATTTGCAATTAAGCAGTTTCTATACTTCACAACCAAAAGCTATCAATCTTGAGAGTCGTTACTTTGATACGATAGAGGGATTC
AAACTTGGAGCAAGTGGAGATAGTTTTGTCCATGACAAGTTCTACATAAATAGATACACTGCCCATGTAAATGACAATCCCGG
AATTACAACTAACAACGTTGATTCAACCAGACTCGTTACAGTTACAGCAACTGGGAATGTAGGAATTGGAACTACTAGTCCAA
GTAAGACACTTGATGTAAATGGCGATGCCATTGCAAATAACTGGCTTAGACATTCCAGCCAGCAATGGAAAACAGACATAGC
ACCACTAGATAACTCCCTAAGCAAGCTAGAGCAGCTACAAGGCATAAGCTACAAGTGGAAAGCAGATGAATTCGCAGACATG
GGATTTCACAATAAAACAACTCTTGGGTTTATTGCCGAAGATGTAGAGACTATACTTCCACAGCTAATCCATACTGACCAAAAT
GGTGAAAAATCAATGGACTATGGTAAACTAACACCAATTCTAGTTGAAGCAATCAAAGAGCAGCAAAACACCATAGAACAGC
TAACCACTGCAATATGTAAAGACAGCCCAAAACATGACGTGTGCAGCAGCTAAAAAACACTTTTTTTTAATTTTTTGATCTTAC
AGTACAACCAGCAGCACTGAAACATTTTTGGCATCGAATCTAAAAAGAAAAAAAAATCATACTATCACATAATGATGATATGC
TGTATTGTGAGAAACTCAAAGACACTGAAAAACATTTTTGGCATCGAATCTAAAAAGAAAAAAAAATCATACTATCACATAAT
GATGATGATGATGCTGTATTGTGAGAAACTCAAAGACACTGAAACATTTTATACAATCCATTAAACTTGAAAACATGACAGGG
CCAACAAATCTCAAAATACTTCCTATATACCTTACACGCCATAA

	CAC	TTC	TCA	TTG	AAA	CGA	CAA	GAT	AGT
	H	F	S	L	K	R	Q	D	S
	AGT	ATT	ACA	CAT	CAG	ACT	GTG	TCA	ATT
	S	I	T	H	Q	T	V	S	I
	TTT	TGC	ATT	ACC	ATC	TTG	CTT	TTA	TCA
	F	C	I	T	I	L	L	L	S
	GTT	AGT	GCA	TAC	TTT	CCT	AAT	TCT	ATT
	V	S	A	Y	F	P	N	S	I
	CCA	GAG	GCG	CAA	GCT				
	P	E	A	Q	A				
	AAG	CTT	GGA	GAT	CAG	CGA	CAG	ATA	GAT
1	K	L	G	D	Q	R	Q	I	D
	ATT	GCA	AGC	TGG	AAC	ACA	TTT	GAT	TTT
	I	A	S	W	N	T	F	D	F
	GGT	GGC	AAG	TTT	GGC	GGT	GTA	TGT	AAG
19	G	G	K	F	G	G	V	C	K
						⇐SOR-Inv2			

Figure S5. Nucleotide sequence and deduced amino acid sequence of full-length natural SOR.

```
nt    1-54     AAG CTT GGA GAT CAG CGA CAG ATA GAT ATT GCA AGC TGG AAC ACA TTT GAT TTT
aa    1-18     K   L   G   D   Q   R   Q   I   D   I   A   S   W   N   T   F   D   F
nt   55-108    GGT GGC AAG TTT GGC GGT GTA TGT AAG GCA AAT GAT CCT ACC ATA CTG CAA GCA
aa   19-36     G   G   K   F   G   G   V   C   K   A   N   D   P   T   I   L   Q   A
nt  109-162    ATG ACT GAT GTA ATA TCT CAG CAT GAT GTC ATT TTG GTT CAA GAG ATT GCA AAT
aa   37-54     M   T   D   V   I   S   Q   H   D   V   I   L   V   Q   E   I   A   N
nt  163-216    GTC ACA AAT CCA TGT AAT CCT GCA AAC ATC TTT TCA GGC TTT AAT AAT TTA TGT
aa   55-72     V   T   N   P   C   N   P   A   N   I   F   S   G   F   N   N   L   C
nt  217-270    TCA AAT AAC TAT CTT GGC GGT CTG GGA TAT CTC TGC CAA GAT ACT GGC ATT GTA
aa   73-90     S   N   N   Y   L   G   G   L   G   Y   L   C   Q   D   T   G   I   V
nt  271-324    GGC TCT GGT GAG GGC TAT GGT ATT ATC TAC AAA AAC AAC ACC GGT ATT GGT AAT
aa   91-108    G   S   G   E   G   Y   G   I   I   Y   K   N   N   T   G   I   G   N
nt  325-378    GTA GTA ATT GAG CGT ACA GAT AAC CCA AAT ACA GTT CCA TAC ATT CCT GCA GGT
aa  109-126    V   V   I   E   R   T   D   N   P   N   T   V   P   Y   I   P   A   G
nt  379-432    GGT GGC AAC AAC CAA ATG AAG AGG CCA CCA ATG AAG GCA ACC CTT GAC TTT AAT
aa  127-144    G   G   N   N   Q   M   K   R   P   P   M   K   A   T   L   D   F   N
nt  433-486    GCA GGT GCA TTC AAC GTA GTA GTA TAT AAT GCA CAC ACA AGC CCA AGT GGC GGT
aa  145-162    A   G   A   F   N   V   V   V   Y   N   A   H   T   S   P   S   G   G
nt  487-540    GTT CCC AAA GAG ATA AAG CTA CTC AGT GAT AGA ATA TCG CCT GCT CAC AAT CCG
aa  163-180    V   P   K   E   I   K   L   L   S   D   R   I   S   P   A   H   N   P
nt  541-594    CTA GAT AAA AAC GCA GTA ATT ACA CTA GGT GAT CTT AAT GCC GAC GGC AAA AGA
aa  181-198    L   D   K   N   A   V   I   T   L   G   D   L   N   A   D   G   K   R
nt  595-648    AGA CTG CCT AAT GGA CAG CTA GAC CAT GGA ACA GAC TAT TAC AGA GGT GGC TTT
aa  199-216    R   L   P   N   G   Q   L   D   H   G   T   D   Y   Y   R   G   G   F
nt  649-702    GCA GCT CAC CCA AAT GAC TTT CCT GTT AGT GGA TGG CAG CAG ACA GTT ACT GAT
aa  217-234    A   A   H   P   N   D   F   P   V   S   G   W   Q   Q   T   V   T   D
nt  703-756    ACT GAT GTT ACA AAC TTT GCA CAG ACA CTT CGT GCA TAT GAT AGA ATG ATA CTT
aa  235-252    T   D   V   T   N   F   A   Q   T   L   R   A   Y   D   R   M   I   L
nt  757-810    AGC AAT GCA ACA CAT AAC TCT TAT GAT GAG TAT GGA ATA ATA GGT GGC ATT CCA
aa  253-270    S   N   A   T   H   N   S   Y   D   E   Y   G   I   I   G   G   I   P
nt  811-864    AAC CCA CTT CCA TAT CCG GGA GGA GTT TAT CCT GCT GGA ACT TTG TTT AAA AAT
aa  271-288    N   P   L   P   Y   P   G   G   V   Y   P   A   G   T   L   F   K   N
nt  865-918    GTA CAT ACT GGC GGT GGA AAT ACA GGA AAG GCA CTC TCT GAT CAT AGA CTG ATA
aa  289-306    V   H   T   G   G   G   N   T   G   K   A   L   S   D   H   R   L   I
nt  919-972    TGG TCA AAG CTT AAC TAT GGT GCA ATA GAG ACT ACC GAT AAT AAC GGC AAT ACA
aa  307-324    W   S   K   L   N   Y   G   A   I   E   T   T   D   N   N   G   N   T
nt  973-1026   AAG TCC AAT TTT GTT GAA TTT ACA AAC CCA CCA CAA TGT ACT GCA CCA GAT ACC
aa  325-342    K   S   N   F   V   E   F   T   N   P   P   Q   C   T   A   P   D   T
nt 1027-1080   GTA TAT GCA AAG GGT GCA GAC TTT GTA CCA AAT AGA ACA GTT GAT ATT TAC ATC
aa  343-360    V   Y   A   K   G   A   D   F   V   P   N   R   T   V   D   I   Y   I
nt 1081-1134   ACA AAG CAG CCA GAC AAT CAA AAC TTT ATC CCA CTG CCA CAG CCA CAA AAC CCA
aa  361-378    T   K   Q   P   D   N   Q   N   F   I   P   L   P   Q   P   Q   N   P
nt 1135-1188   ACA AAC CCT GGG CAG TTC AAG CAA GTT GGC GAA TCA TAC CAG CTA ACA GAT GTA
aa  379-396    T   N   P   G   Q   F   K   Q   V   G   E   S   Y   Q   L   T   D   V
nt 1189-1242   AGG ACT ACA GGC AAA AGT ACG CAC AAC ATT GAT GCA AAC GGC AAC ATT GCA AAT
aa  397-414    R   T   T   G   K   S   T   H   N   I   D   A   N   G   N   I   A   N
nt 1243-1296   ACT GCT GTA TGG GTA CAG CCA ACA CCA AAC CAG CAG TAC AAC CTC ATA GTT GAT
aa  415-432    T   A   V   W   V   Q   P   T   P   N   Q   Q   Y   N   L   I   V   D
nt 1297-1350   GTG GAC CGT GAT GGA TAC TTT ACA AAC AGC ATA GAT ATA GTT GAT GCA TTT AAC
aa  433-450    V   D   R   D   G   Y   F   T   N   S   I   D   I   V   D   A   F   N
nt 1351-1404   ACA CAA GGC TTT GAT GTA GAC CCA TGC CTT CGA AAG AGA CTA GCA GCA TCA CTT
aa  451-468    T   Q   G   F   D   V   D   P   C   L   R   K   R   L   A   A   S   L
nt 1405-1458   TCT GAT GGA ATA CCA GCA GGC GAT GGC ACA CGT GAC TTT GTA AAA CCT CCC AAA
aa  469-486    S   D   G   I   P   A   G   D   G   T   R   D   F   V   K   P   P   K
nt 1459-1512   ATA TTT ATT TCC GGT GAC TGG TTC CCA CAG GGT TCT GCA CGT ATC TAC ATT ATC
aa  487-504    I   F   I   S   G   D   W   F   P   Q   G   S   A   R   I   Y   I   I
nt 1513-1566   ATA TAT GAC AAG AAC TTT GAT TAT AAC GGC AAT GAC TCC AAA GTA TTA ACT GAC
aa  505-522    I   Y   D   K   N   F   D   Y   N   G   N   D   S   K   V   L   T   D
nt 1567-1620   GTC CGT GGA ACT TAT GAC ACA ATT ACA GTG GGT GAG AAC CAC AAG TTT ACA GGA
aa  523-540    V   R   G   T   Y   D   T   I   T   V   G   E   N   H   K   F   T   G
nt 1621-1674   CTA GAA TGG TCC TCA CCA CAT GTT GGT AAC TAT AAC ATA ATA TTT GAT GCA AAC
aa  541-558    L   E   W   S   S   P   H   V   G   N   Y   N   I   I   F   D   A   N
nt 1675-1728   TCT GAT GGA GTA TAC AAA AAA TCC GAT GGT GAC ATT GTA GAT CAC ATA AAC GAG
aa  559-576    S   D   G   V   Y   K   K   S   D   G   D   I   V   D   H   I   N   E
nt 1729-1782   ATA GGC TTT GCA ATG CTC GAA TCG TAT CCA CAT CAC AGC CAT ATT GTA GAT TTA
aa  577-594    I   G   F   A   M   L   E   S   Y   P   H   H   S   H   I   V   D   L
nt 1783-1836   GAT GAC AAT GCA AAC CAG CGA GAA GAC TTT AAC AGT GGA ATT GCA AAA AAC GTC
aa  595-612    D   D   N   A   N   Q   R   E   D   F   N   S   G   I   A   K   N   V
```

nt 1837-1890 TAC GTT CTT GCA ACT AAT CTT CCA AAC CCA AGC ACA ATA GTT GAC GTT TAT GTA
aa 613-630 Y V L A T N L P N P S T I V D V Y V

nt 1891-1944 ATT AGT GAG AAA CTC TTA AAG CTA AAC AAT CCT GGA TGG ACC ACA TGG CAG CAA
aa 631-648 I S E K L L K L N N P G W T T W Q Q

nt 1945-1998 TCC TCT AAT GTG CCA CTA AAG GAG AGT GCA GCA CCA ACT AAC AAT GGA GAC AGG
aa 649-666 S S N V P L K E S A A P T N N G D R

nt 1999-2052 CTA CAC CCT ACT TGG GTA ACT CCA CAA AAG ACA GTA TTT GGT TCA GTA TGG CAT
aa 667-684 L H P T W V T P Q K T V F G S V W H

nt 2053-2106 ATG CCC GGT GAT ACA TTT GAT CCA CCA TAC ATT AAT TAT TAC GGC AAA AAA TTC
aa 685-702 M P G D T F D P P Y I N Y Y G K K F

nt 2107-2160 ACT ATA GTA ATT GAT GTG AAT CGT GAT GGT AAG TAT GAT CCT AGT ATA GAT ATG
aa 703-720 T I V I D V N R D G K Y D P S I D M

nt 2161-2214 GTA GAT ACT CAC GAT ATA GGA GAC ATG ACA GAC TGG TTT ATC ACA CAG GGT CAC
aa 721-738 V D T H D I G D M T D W F I T Q G H

nt 2215-2268 CAT GAC CTA AGC CCA ACT AGT GGA AAC GGA CAG CCT GCC GTA TCT GAA TAC AAA
aa 739-756 H D L S P T S G N G Q P A V S E Y K

nt 2269-2322 GAG TAT CTC AAC TCA AAG CTA GAC TTG GAG AAC CCG CTT GAT AAC ACC AAC AAC
aa 757-774 E Y L N S K L D L E N P L D N T N N

nt 2323-2376 TAT GAT GCT GCA ACT GAG GCA GCT TCT GCA CAA TAT CTA TGC CGT ACC AAT CTT
aa 775-792 Y D A A T E A A S A Q Y L C R T N L

nt 2377-2430 CCA ATC AAT CTA TTT GAG AAT GTC ATA GAG CTG GGA GCT CAG ACA GGC TTT ATC
aa 793-810 P I N L F E N V I E L G A Q T G F I

nt 2431-2484 ATG TTT AAC CCT GAT GAG TAT TAT GCA GGC CGT GAT CTA AAT TCT GGA CGC CAC
aa 811-828 M F N P D E Y Y A G R D L N S G R H

nt 2485-2538 ATT TAT GAT GCT GTA GAT TTT GAT GGC TTG GAG ATA AAT GAC GGA TAC ACA TCA
aa 829-846 I Y D A V D F D G L E I N D G Y T S

nt 2539-2592 GTA ATC TCT GCA AAA GAG ATG ACC CTA AAC AGA TTA GGT GTT GGC CAA AAC TCC
aa 847-864 V I S A K E M T L N R L G V G Q N S

nt 2593-2646 AAT CTG CTT ACC TGC GGA GCT GAC ACA GTA ACG ATA GAT GGC ATG TCA ACA CAA
aa 865-882 N L L T C G A D T V T I D G M S T Q

nt 2647-2700 AAA GAT TCA ACT ACA AAT ATT GTT GCA AAG ACC ATA TCT CAC AAA AGT AAC ATC
aa 883-900 K D S T T N I V A K T I S H K S N I

nt 2701-2754 TCA CTA GAG GCA GAC ACT TGT ACA CAT TAT ACT CAC AAT CTC TAC TTT GAG TCA
aa 901-918 S L E A D T C T H Y T H N L Y F E S

nt 2755-2808 GAC ACC AAC ATC TTA GTT AAG GCC ATT GAG AGT CCA TTT GCA TGG ATA ATT GAC
aa 919-936 D T N I L V K A I E S P F A W I I D

nt 2809-2844 GTA GGT GAC AGA ATA CTT CCA GGT ACT GGC TGT TAG
aa 937-948 V G D R I L P G T G C *

Figure S6. Alphafold-3 followed by Foldseek analysis of SOR and its domains.

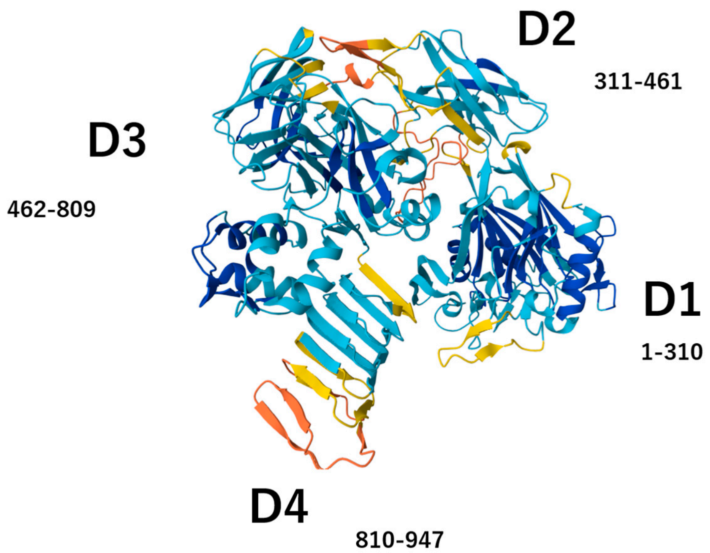

Representative structural homologs identified by Foldseek analysis of SOR domains 2–4.

SOR domain	Target accession	Annotation	Query region	Sequence identity (%)	E-value
**D2**	**A0A235BR23**	Ig-like domain-containing protein	2–148	23.8	3.26×10^{-8}
D2	**A0A098ECD4**	Probable pectate lyase C	1–148	27.7	2.05×10^{-7}
D2	**A0A662RJX4**	Carbohydrate-binding module family 96 domain-containing protein	3–151	22.1	2.41×10^{-7}
**D3 (462–808)**	**A0A8J6LX49**	Endonuclease/exonuclease/phosphatase family protein	3–347	29.2	8.28×10^{-20}
D3 (462–808)	**A0A9E3EYY6**	Endonuclease/exonuclease/phosphatase domain-containing protein	22–347	33.5	7.72×10^{-19}
D3 (462–808)	**A0A662U062**	VCBS repeat-containing protein (fragment)	2–260	20.2	2.11×10^{-13}
**D4**	**A0A7L1YC00**	AHNK protein (fragment)	10–137	8.3	9.50×10^{-2}
D4	**A0A3M2L7X1**	Helix-turn-helix domain-containing protein	10–138	10.6	2.06×10^{-1}
D4	**A0A1I7XI96**	DUF4367 domain-containing protein	10–138	12.2	2.57×10^{-1}

Structural similarity searches were performed using Foldseek against the AlphaFold Database (AFDB50). Representative top-ranked hits are shown. Although domains 2 and 3 showed significant structural similarities to several proteins, these similarities do not necessarily imply equivalent biological functions. Domain 4 did not yield a strongly significant structural homolog.

Figure S7. Expression of recombinant SOR and ECP in *Escherichia coli.*

A. Expression of rSOR in *E. coli.*

Lanes: M, molecular weight marker; 1, control bacterial pellet; 2, bacterial pellet expressing rSOR; 3, control cell extract; and 4, cell extract expressing rSOR. These data show that the expressed protein was obtained only in the pellet.

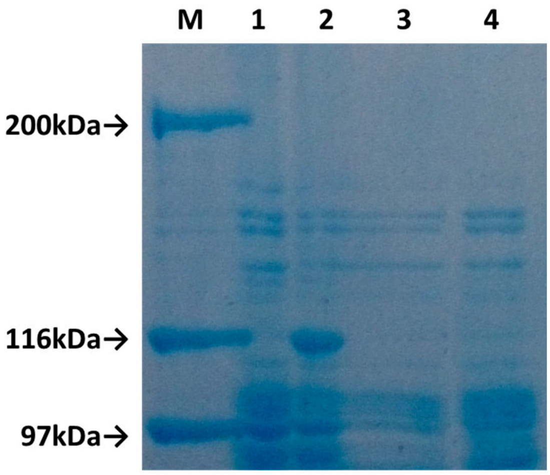

B. Expression of ECP constructs in *E. coli.*

Lanes: M, molecular weight marker; 1, control bacterial pellet; 2, bacterial pellet expressing rEC1; 3, bacterial pellet expressing rEC2; 4, control cell extract; 5, cell extract expressing rEC1; and 6, cell extract expressing rEC2. These data show that the expressed protein was obtained only in the pellet. Note that rEC1 include N-terminal propeptide and rEC2 starts from deduced N-terminal sequence.

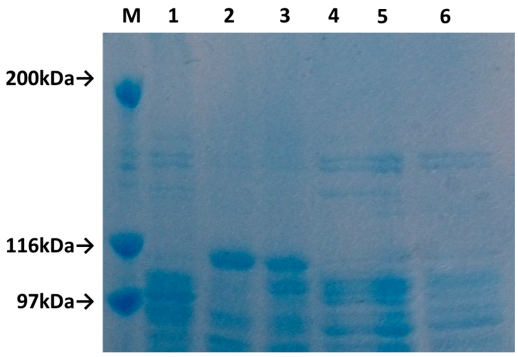

Figure S8. Expression and purification of N-SOR.

A. Codon-optimized nucleotide sequence encoding the N-terminal 310 amino acids of SOR. The 930-nt construct translates exactly to residues 1–310 of the full-length SOR protein.

```
nt     1-54     AAG CTC GGC GAT CAA CGT CAA ATC GAT ATC GCG AGC TGG AAT ACG TTT GAC TTC
aa     1-18     K   L   G   D   Q   R   Q   I   D   I   A   S   W   N   T   F   D   F
nt    55-108    GGT GGG AAA TTC GGC GGC GTG TGC AAA GCC AAT GAC CCG ACG ATT CTC CAA GCG
aa    19-36     G   G   K   F   G   G   V   C   K   A   N   D   P   T   I   L   Q   A
nt   109-162    ATG ACT GAT GTG ATC TCC CAG CAC GAT GTA ATT CTG GTC CAG GAG ATC GCC AAC
aa    37-54     M   T   D   V   I   S   Q   H   D   V   I   L   V   Q   E   I   A   N
nt   163-216    GTG ACG AAC CCG TGT AAC CCC GCT AAT ATT TTC AGT GGC TTC AAT AAC TTG TGT
aa    55-72     V   T   N   P   C   N   P   A   N   I   F   S   G   F   N   N   L   C
nt   217-270    AGT AAC AAC TAC TTA GGT GGT TTA GGT TAT CTT TGC CAA GAC ACT GGG ATC GTT
aa    73-90     S   N   N   Y   L   G   G   L   G   Y   L   C   Q   D   T   G   I   V
nt   271-324    GGT AGC GGT GAG GGC TAT GGG ATC ATC TAC AAG AAC AAT ACC GGT ATC GGT AAC
aa    91-108    G   S   G   E   G   Y   G   I   I   Y   K   N   N   T   G   I   G   N
nt   325-378    GTC GTC ATC GAG CGC ACT GAT AAC CCG AAT ACT GTG CCA TAT ATT CCC GCC GGA
aa   109-126    V   V   I   E   R   T   D   N   P   N   T   V   P   Y   I   P   A   G
nt   379-432    GGC GGC AAC AAC CAG ATG AAG CGC CCG CCG ATG AAG GCA ACG CTT GAT TTC AAT
aa   127-144    G   G   N   N   Q   M   K   R   P   P   M   K   A   T   L   D   F   N
nt   433-486    GCG GGC GCC TTC AAT GTA GTC GTG TAT AAT GCC CAC ACG AGC CCA TCG GGC GGC
aa   145-162    A   G   A   F   N   V   V   V   Y   N   A   H   T   S   P   S   G   G
nt   487-540    GTA CCA AAG GAA ATC AAA TTG CTC TCC GAC CGT ATC AGC CCA GCA CAT AAT CCT
aa   163-180    V   P   K   E   I   K   L   L   S   D   R   I   S   P   A   H   N   P
nt   541-594    CTT GAC AAG AAC GCA GTA ATT ACT TTG GGA GAC TTG AAT GCG GAT GGA AAG CGT
aa   181-198    L   D   K   N   A   V   I   T   L   G   D   L   N   A   D   G   K   R
nt   595-648    CGC CTG CCA AAT GGA CAG TTA GAT CAC GGT ACA GAT TAT TAC CGC GGC GGA TTT
aa   199-216    R   L   P   N   G   Q   L   D   H   G   T   D   Y   Y   R   G   G   F
nt   649-702    GCC GCA CAT CCG AAC GAC TTT CCG GTA AGT GGG TGG CAG CAA ACT GTG ACG GAT
aa   217-234    A   A   H   P   N   D   F   P   V   S   G   W   Q   Q   T   V   T   D
nt   703-756    ACT GAT GTA ACC AAC TTC GCA CAA ACT CTC CGT GCG TAC GAC CGC ATG ATC TTA
aa   235-252    T   D   V   T   N   F   A   Q   T   L   R   A   Y   D   R   M   I   L
nt   757-810    TCA AAC GCC ACG CAC AAT AGT TAC GAC GAG TAT GGG ATC ATC GGC GGC ATC CCT
aa   253-270    S   N   A   T   H   N   S   Y   D   E   Y   G   I   I   G   G   I   P
nt   811-864    AAC CCT CTC CCC TAC CCG GGT GGC GTT TAC CCC GCC GGA ACG TTG TTC AAG AAC
aa   271-288    N   P   L   P   Y   P   G   G   V   Y   P   A   G   T   L   F   K   N
nt   865-918    GTG CAT ACA GGC GGT GGC AAC ACA GGA AAG GCT CTC AGT GAT CAC CGT CTG ATC
aa   289-306    V   H   T   G   G   G   N   T   G   K   A   L   S   D   H   R   L   I
nt   919-930    TGG TCT AAG TTA
aa   307-310    W   S   K   L
```

B. SDS-PAGE of expression product

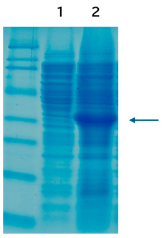

Expression Products
1. Supernatant
2. Precipitate

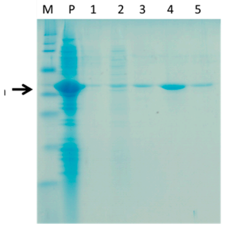

Ni column purification

P: precipitation
1˜5 eluted fractions

Figure S9. Bioactivity of SOR and FITC-SOR used in this study.

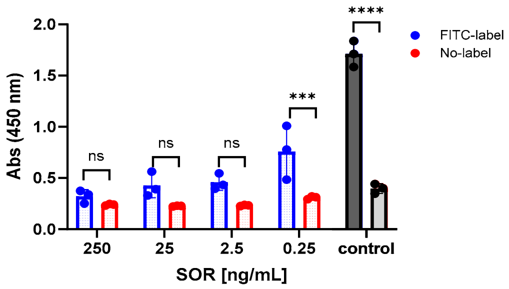

Figure S10. Fluorescent micrographs of HeLa cells treated with FITC-SOR.

Cells were preincubated at 4°C or 37°C for 15 min before the addition of FITC-SOR (50 μg/mL). After incubation with FITC-SOR for 1 h at the respective temperatures, the cells were fixed with methanol. Image brightness was adjusted separately for each condition for visualization, because the intense cell-surface fluorescence at 4°C was saturated under the display settings used for the 37°C images. Therefore, fluorescence intensities between the 4°C and 37°C images should not be directly compared.

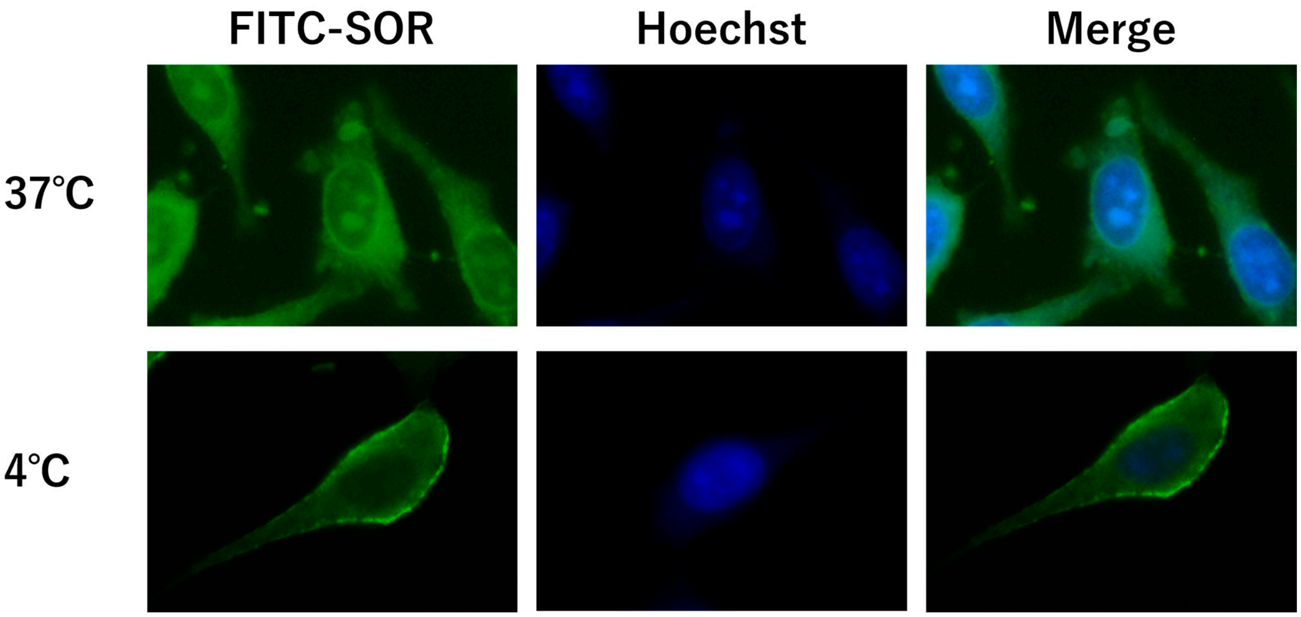

Figure S11. Proportions of immunostained cells at 2 and 6 h after SOR treatment.

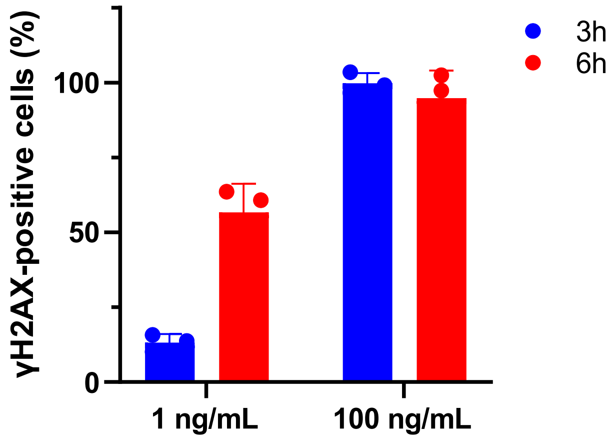

Supplement: Supplementary file 1 [file toxins-18-00434-s001.zip › toxins-4502007-supp.pdf]
